# Electrosynthesis of Biocompatible Free-Standing PEDOT Thin Films at a Polarized Liquid|Liquid Interface

**DOI:** 10.1021/jacs.1c12373

**Published:** 2022-03-09

**Authors:** Rob A. Lehane, Alonso Gamero-Quijano, Sigita Malijauskaite, Angelika Holzinger, Michele Conroy, Fathima Laffir, Amit Kumar, Ursel Bangert, Kieran McGourty, Micheál D. Scanlon

**Affiliations:** † Bernal Institute, University of Limerick (UL), Limerick V94 T9PX, Ireland; ‡ Department of Chemical Sciences, School of Natural Sciences, University of Limerick (UL), Limerick V94 T9PX, Ireland; § Department of Physics, School of Natural Sciences, University of Limerick (UL), Limerick V94 T9PX, Ireland; ∥ School of Mathematics and Physics, Queen’s University Belfast (QUB), Belfast BT71 NN, UK; ⊥ Health Research Institute (HRI), University of Limerick (UL), Limerick V94 T9PX, Ireland; # The Advanced Materials and Bioengineering Research (AMBER) Centre, CRANN Institute, Trinity College Dublin (TCD), Dublin 2 D02 PN40, Ireland

## Abstract

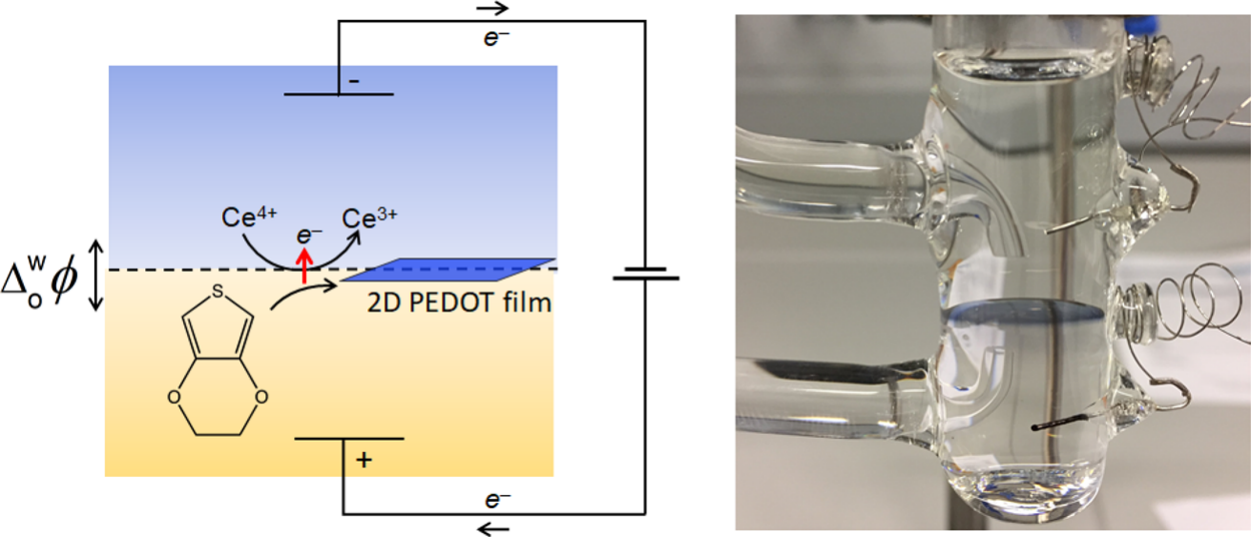

Conducting polymers (CPs) find applications in energy conversion and storage, sensors, and biomedical technologies once processed into thin films. Hydrophobic CPs, like poly(3,4-ethylenedioxythiophene) (PEDOT), typically require surfactant additives, such as poly(styrenesulfonate) (PSS), to aid their aqueous processability as thin films. However, excess PSS diminishes CP electrochemical performance, biocompatibility, and device stability. Here, we report the electrosynthesis of PEDOT thin films at a polarized liquid|liquid interface, a method nonreliant on conductive solid substrates that produces free-standing, additive-free, biocompatible, easily transferrable, and scalable 2D PEDOT thin films of any shape or size in a single step at ambient conditions. Electrochemical control of thin film nucleation and growth at the polarized liquid|liquid interface allows control over the morphology, transitioning from 2D (flat on both sides with a thickness of <50 nm) to “Janus” 3D (with flat and rough sides, each showing distinct physical properties, and a thickness of >850 nm) films. The PEDOT thin films were *p*-doped (approaching the theoretical limit), showed high π–π conjugation, were processed directly as thin films without insulating PSS and were thus highly conductive without post-processing. This work demonstrates that interfacial electrosynthesis directly produces PEDOT thin films with distinctive molecular architectures inaccessible in bulk solution or at solid electrode–electrolyte interfaces and emergent properties that facilitate technological advances. In this regard, we demonstrate the PEDOT thin film’s superior biocompatibility as scaffolds for cellular growth, opening immediate applications in organic electrochemical transistor (OECT) devices for monitoring cell behavior over extended time periods, bioscaffolds, and medical devices, without needing physiologically unstable and poorly biocompatible PSS.

## Introduction

Conducting polymers (CPs) find widespread applications in energy conversion and storage, sensors, and optoelectronic, photovoltaic, bioelectronic, and biomedical technologies.^1−6^ To satisfy this diverse set of applications, fabrication routes to CPs with desirable shapes/dimensions and compatibility with any substrate are required. The lightweight, flexible, and transparent nature of CPs makes them ideal for incorporation into technologies as thin films.^7^

Current methodologies to generate CP thin films have deficiencies that hinder technological progress. For example, multistep film processing methods following chemical polymerization require a complex of the CP and a hydrophilic surfactant, typically poly(styrenesulfonate) (PSS), to aid the processability of technologically ubiquitous hydrophobic CPs, such as poly(3,4-ethylenedioxythiophene) (PEDOT).^8^ However, excess PSS diminishes CP conductivity, long-term stability, specific capacity, and biocompatibility.^9−12^ Electropolymerization produces CP thin films in a single step^13^ but is limited to deposition onto conducting surfaces and is less easily adapted to the synthesis of composite materials.^14^ Vapor phase techniques are capable of producing CP thin films with record-high electrical conductivity^15^ but require high vacuum and/or high temperatures can be complicated or expensive to implement and incompatible with heat-sensitive substrates.^16^

A liquid|liquid (L|L) interface provides a reproducible and defect-free environment to prepare and process free-standing two-dimensional (2D) thin films of nanomaterials, such as CPs, in a single step.^17^ By polarizing certain L|L interfaces known as interfaces between two immiscible electrolyte solutions (ITIES),^18−20^ exquisite electrochemical control over the kinetics of interfacial electron transfer (IET) between oxidant and monomer redox couples in opposite phases is achieved.

The groups of Cunnane, Mareček, and Dryfe provided early insights into electrosynthesis of conducting polymers (CPs) at polarized aqueous|organic interfaces.^21−28^ Cunnane first reported the electropolymerization of short oligomer chains using 1-methylpyrrole and 1-phenylpyrrole monomers.^22^ However, no interfacial thin film was formed with these relatively hydrophilic oligomers. Analysis of CP films formed with 2,2′:5′,2″-terthiophene showed poor electrochemical stability and conductivity,^24^ likely due to overoxidation of any CPs formed as the aqueous oxidant was consistently in significant excess over the monomer. Dryfe’s group grew a polypyrrole film on single-walled carbon nanotubes at the interface during electropolymerization.^28^ However, the relatively hydrophilic pyrrole partitions to the aqueous phase, leading to uncontrolled chemical polymerization in that phase. Electrosynthesis of Janus-type gold/CP composites has been reported by Nishi et al. at a polarized L|L interface formed between a hydrophobic ionic liquid and water.^29^ Upscaling biphasic ionic liquid-based systems will be challenging due to their excessive viscosity and low conductivity.

In this work, we report a major advance in the use of interfacial electrosynthesis at an ITIES to prepare free-standing, additive-free, reproducible, easily transferrable, scalable, and biocompatible PEDOT thin films in a single step at optimized ambient conditions. The external electrochemical driving force, the interfacial Galvani potential difference (Δ_o_^w^ϕ), is applied using double-potential step chronoamperometry (DPSCA). The IET reaction between an aqueous Ce^4+^ oxidant and organic EDOT monomer and subsequent oligomer deposition at the L|L interface can be assisted or hindered by the manipulation of Δ_o_^w^ϕ. We demonstrate an emergent enhanced biocompatibility of free-standing PEDOT thin films prepared at the ITIES, compared with films prepared by drop-casting commercial PEDOT:PSS surfactant-free ink, using normal human retina pigment epithelium (hTERT RPE-1) cells. The latter are a physiologically pertinent cell line given the application of PEDOT films in maculopathies.^30,31^ We functionally modified the PEDOT and PEDOT:PSS films through the incorporation of bioactive proteins and monitored the consequential effects on cell behavior in each case. These findings foreshadow potential applications as suitable 2D conductive substrates for RPE and electrically active photoreceptor cells and the development of organic electrochemical cell transistors (OECTs) capable of electrochemically monitoring cell growth over >24 h periods.

## Results and Discussion

### Mechanism of PEDOT Interfacial Electrosynthesis

**Figure 1:**
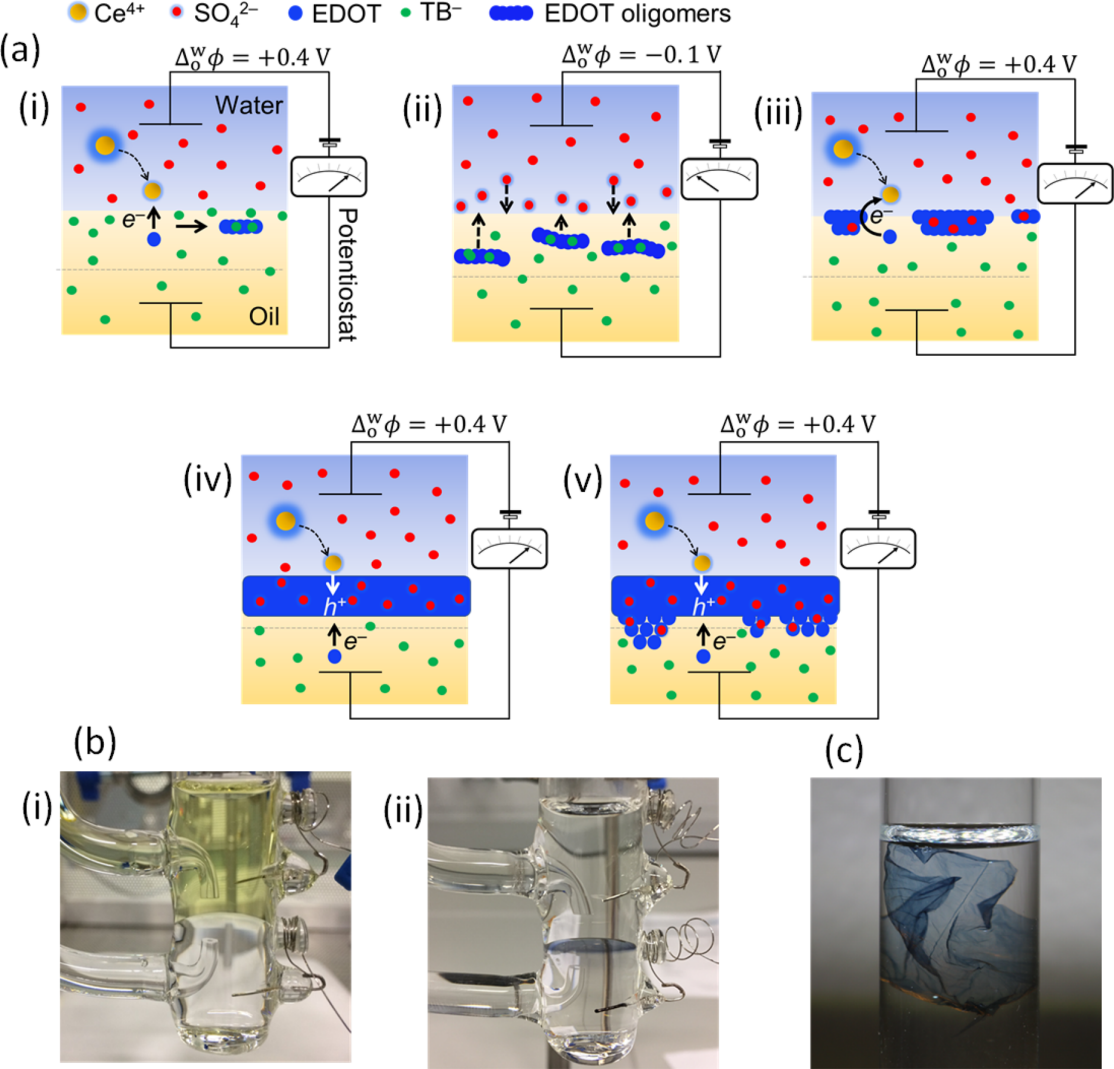
The mechanism of PEDOT interfacial electrosynthesis at the interface between two immiscible electrolyte solutions (ITIES). (a) The mechanism is schematically shown as five distinct steps with time: (i) interfacial electron transfer (IET) at a positive externally applied interfacial Galvani potential difference (Δ_o_^w^ϕ = +0.4 V) between the aqueous Ce^4+^ oxidant and organic EDOT monomer to form cationic EDOT oligomers, (ii) interfacial adsorption of the cationic EDOT oligomers at a more negative Δ_o_^w^ϕ (−0.1 V) through an ion-pairing and interchange interaction with the aqueous SO_4_^2 –^ anions, (iii) autocatalytic IET between Ce^4+^ and EDOT at adsorbed EDOT oligomer sites that act as interfacial bipolar electrodes, (iv) adsorbed EDOT oligomer coalescence to form a highly compact 2D PEDOT thin film at the ITIES that is flat on both sides and heavily doped with aqueous SO_4_^2–^ anions, and (v) continued IET leading to a secondary growth process into the organic phase and the formation of a porous 3D structure on the organic-facing side as the thickness of the PEDOT thin film increases. (b) Four-electrode electrochemical cell (i) before and (ii) after interfacial electrosynthesis. The acidic aqueous phase, containing the yellow Ce^4+^ oxidant, is on top and the more dense α, α, α-trifluorotoluene (TFT) organic solution containing the EDOT monomer is on the bottom. PEDOT forms exclusively as a thin blue film at the polarized liquid|liquid (L|L) interface. (c) A PEDOT thin film removed from a large ITIES and stored in an acetone/0.2 M H_2_SO_4_ mixture to minimize gradual undoping.

As illustrated in Figure 1ai–v, PEDOT thin film interfacial electrosynthesis progresses along five distinct stages with time. Initially, IET occurs between the aqueous Ce^4+^ oxidant and EDOT organic monomer, forming monomeric radical cations (EDOT^·+^) in the diffusion layer on the organic side of the ITIES (Figure 1ai). For IET to proceed with appreciable kinetics, the ITIES must be polarized positively as detailed in the Supporting Information and discussed vide infra, with Δ_o_^w^ϕ set to a value near the positive extreme of the Galvani polarizable potential window. EDOT^·+^ species are stabilized by the weakly coordinating organic anion, tetrakis(pentafluorophenyl)borate (TB^–^),^32^ and further couple with each other or with EDOT monomers to form dimers in the diffusion layer. Continuous EDOT^·+^ generation by IET and ensuing radical coupling steps ultimately lead to the formation of EDOT oligomers that also coordinate with TB^–^ to maintain electroneutrality (Figure 1ai). The charge on the bulky TB^–^ anion is primarily centered on the boron atom.^33^ Thus, weakly coordinating TB^–^ has a poor ability to compensate the positive charge on the cationic EDOT oligomers which consequently maintain a net positive charge. The radical coupling steps also result in the release of protons on the organic side of the ITIES (see the Supporting Information and Figure S1) that will be stabilized by the PEDOT thin film itself or by water present inside the film.^34^

Interfacial adsorption involving ion-pairing and interchange between the cationic EDOT oligomers and aqueous electrolyte anions (herein SO_4_^2–^) takes place once the oligomers reach a critical size after an induction period (Figure 1aii). SO_4_^2–^ anions displace the weakly coordinating organic TB^–^ anions during interfacial adsorption, ultimately becoming the sole dopant anion in the PEDOT thin film. This deposition process is driven by the energetically favorable reduction of the interfacial tension (γ) between the two repulsive phases upon oligomer adsorption.^35^ Setting Δ_o_^w^ϕ slightly negative of the potential of zero charge (PZC) is optimal for oligomer adsorption, ensuring the presence of a sufficient concentration of SO_4_^2–^ at the L|L interface to participate in ion-pairing. Thus, as the IET and oligomer interfacial adsorption steps take place at different applied Δ_o_^w^ϕ, potentiodynamic or multistep potentiostatic electrochemical techniques are favored over single-step potentiostatic ones. Also, a negative Δ_o_^w^ϕ will facilitate the pumping of protons, generated on the organic side of the ITIES during the IET/radical coupling process at a positive Δ_o_^w^ϕ, to the aqueous phase via either direct ion transfer or the Grotthuss mechanism through the PEDOT thin film (Figure S2).

In the next step, nucleation and growth of adsorbed EDOT oligomers takes place at the interface. The EDOT oligomers act as floating interfacial bipolar electrodes, providing abundant catalytic sites as electrical shortcuts to catalyze IET between the Ce^4+^ and EDOT species (Figure 1aiii).^36−38^ Due to this autocatalytic effect, IET proceeds at a much lower overpotential than at a bare ITIES with a higher kinetic rate. Thus, the PEDOT islands show rapid 2D growth parallel to the L|L interface. The gaps between individual rapidly growing islands of PEDOT disappear, and a highly compact 2D PEDOT thin film coalesces at the ITIES that is flat on both sides, with a thickness of ∼50 nm (Figure 1aiv). At this point, a physical barrier now exists between the Ce^4+^ and EDOT species at the ITIES. However, IET continues through the conductive PEDOT thin film and is subject to the influence of the diffusion of SO_4_^2–^ counteranions through the film to maintain electroneutrality locally, i.e., “*p*-doping.” At this point, after the PEDOT thin film has coated the interface, the mechanism of SO_4_^2–^ doping can be considered analogous to facilitated ion transfer (FIT) at the polarized L|L interface, specifically the transfer by interfacial complexation (TIC) mechanism.^39−41^ Continued IET initiates a secondary 3D growth process into the organic phase as the thickness of the PEDOT thin film increases. This controllable secondary growth process leads to the formation of a highly porous 3D structure up to ∼850 nm thick (Figure 1av).

### Electrochemically Initiating and Controlling PEDOT Thin Film Interfacial Electrosynthesis

IET between Ce^4+^ and EDOT leading to a 2D PEDOT thin film is not a spontaneous process at an aqueous|α, α, α-trifluorotoluene (TFT) interface (Figure S3). To initiate CP thin film formation, the aqueous|TFT interface must be polarized using a potentiostat in conjunction with a four-electrode electrochemical cell (Figure 1b and Figure S4). The resulting free-standing 2D PEDOT thin film could be recovered from the L|L interface, washed with acetone, and suspended in an acetone:sulfuric acid mixture (Figure 1c) for storage before ex situ characterization or applications. Optimal conditions for thin film formation required a low concentration of oxidant (with the monomer always in excess), which is unconventional in chemical synthesis. A thermodynamic analysis of this biphasic system explains the need for an external electrochemical driving force to drive interfacial electrosynthesis with significant kinetics (Supporting Information).

**Figure 2:**
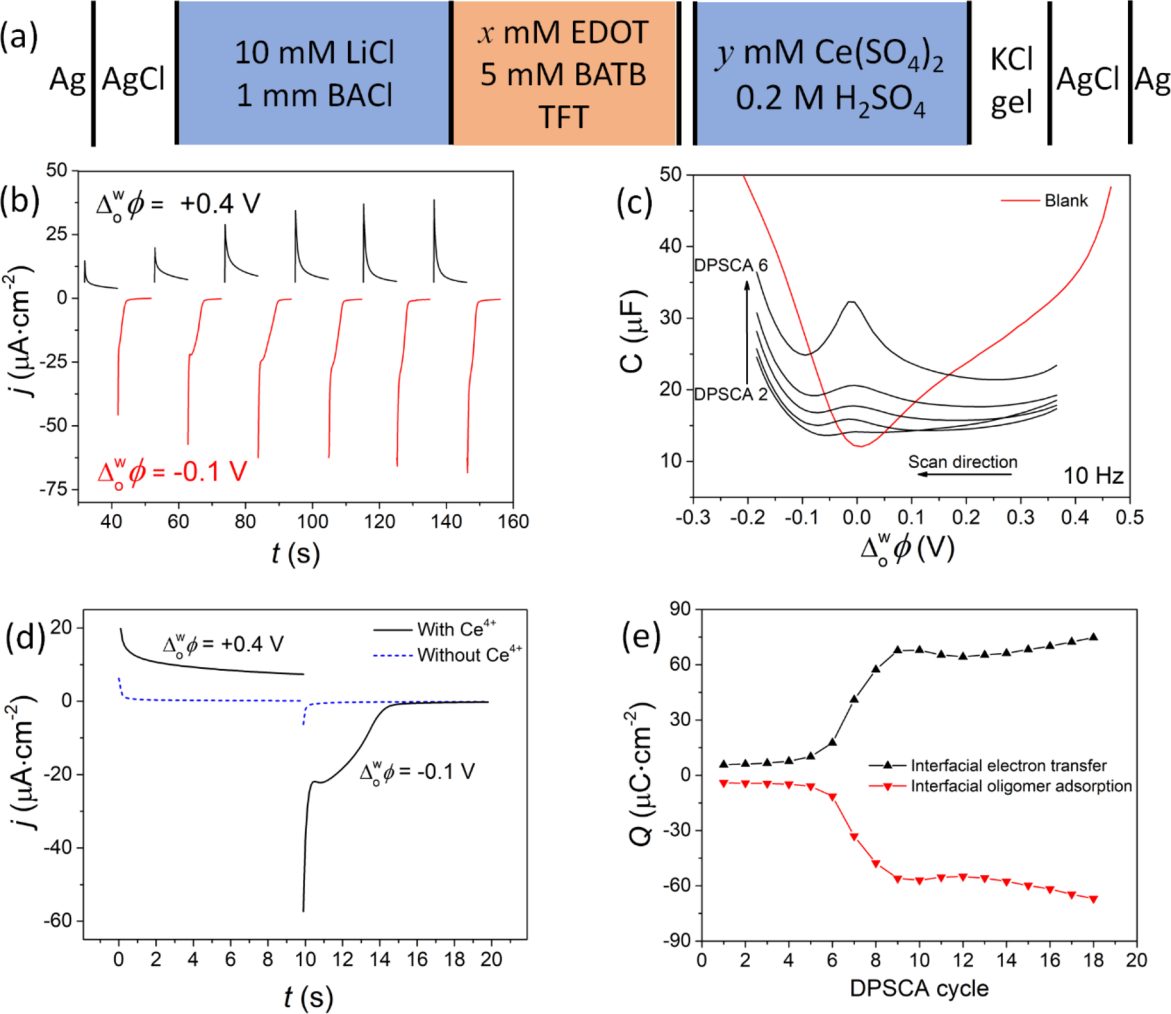
Electrochemically initiating, controlling, and monitoring PEDOT thin film interfacial electrosynthesis. (a) Electrochemical cell configuration of the four-electrode electrochemical cell employed. For blank experiments *x* and *y* are both 0 mM. For interfacial electrosynthesis experiments, *x* and *y* are 5 and 2 mM, respectively. In this four-electrode configuration, the organic Pt and Ag/AgCl electrodes were connected to the counter and reference terminals, respectively, while the aqueous Pt and Ag/AgCl electrodes were connected to the working and sensing terminals, respectively. All experiments were carried out under aerobic conditions. (b) Current–time transients recorded during double-potential step chronoamperometry (DPSCA) cycles 5 to 10 in the presence of aqueous Ce^4+^ and organic EDOT. The first potential step was held at Δ_o_^w^ϕ = +0.4 V for 10 s, and the second step was held at Δ_o_^w^ϕ = −0.1 V for 10 s. (c) Differential capacitance (C/μF) measurements performed at a bare aqueous|TFT interface (red line) and after DPSCA cycles 2 to 6 (black lines) in the presence of aqueous Ce^4+^ and organic EDOT. The frequency was 10 Hz, the amplitude was 10 mV, and the scan direction was from positive to negative potential. (d) Control DPSCA experiments. Current–time transients recorded during a DPSCA cycle with (black lines) and without (dashed blue lines) the aqueous Ce^4+^ oxidation present during PEDOT interfacial electrosynthesis. (e) Plot of the charge (Q/μC·cm^–2^) for each potential step recorded for the first 18 DPSCA cycles. All electrochemical experiments were performed using the cell configuration outlined in panel (a) under aerobic conditions.

Double-potential step chronoamperometry (DPSCA) cycling provided the external driving force, with the four-electrode electrochemical cell configuration outlined in Figure 2a. Current–time transients for the initial six DPSCA cycles are shown in Figure 2b. A single DPSCA cycle involves first holding the Δ_o_^w^ϕ at +0.4 V for 10 s to induce IET between Ce^4+^ and EDOT with appreciable kinetics, leading to a positive current–time transient and the formation of cationic EDOT oligomers in the diffusion zone on the organic side of the ITIES. Next, the Δ_o_^w^ϕ is held at −0.1 V for a further 10 s to induce EDOT oligomer interfacial adsorption, leading to a negative current–time transient. The value of −0.1 V was chosen as it is slightly negative of the PZC at a bare aqueous|TFT interface, determined as ca. 0 V by AC voltammetry (Figure 2c), and optimal for oligomer adsorption as discussed vide supra. After 50 DPSCA cycles, a blue PEDOT thin film becomes visible at the aqueous|TFT interface (Figure 1bii).

No change in charge was observed after 100 DPSCA cycles during control experiments without Ce^4+^ present (Figure 2d). Trends in the kinetics of interfacial electrosynthesis with DPSCA cycle number were determined by analyzing the charge magnitudes recorded from both the positive and negative current transients during the first 18 DPSCA cycles (Figure 2e). These trends corroborated the mechanism outlined in Figure 1a, as detailed in the Supporting Information.

Evidence of interfacial ion-pairing and interchange between adsorbed EDOT oligomers and SO_4_^2–^ anions during interfacial electrosynthesis is provided by the comparison of differential capacitance measurements of the blank aqueous|TFT interface and after DPSCA cycles 2 to 6 in the presence of Ce^4+^ and EDOT (Figure 2c and Supporting Information). In situ parallel-beam UV–Vis absorbance measurements show the depletion of Ce^4+^ on the aqueous side of the L|L interface with continuous DPSCA cycling and PEDOT thin film formation at the ITIES after 300 DPSCA cycles (Figure S11).

Although hydrophobic, the EDOT monomer is slightly soluble in aqueous solutions. Therefore, it is expected that a quantity of the EDOT monomer (and indeed low molecular weight EDOT oligomers) will partition across the L|L interface during the initial stages of the electrosynthesis. This is evident from the strong Tyndall effect seen in the aqueous phase after interfacial electrosynthesis (Figure S12) due to the presence of EDOT oligomers. Definitively distinguishing a “truly heterogeneous IET” mechanism from an “interfacial partition followed by homogeneous ET” mechanism experimentally at a polarized L|L interface is difficult.^42^ As a result, we cannot decisively conclude that the initial IET reaction between Ce^4+^ and the EDOT monomer is truly heterogeneous (shown in Figure 1ai). Nevertheless, partitioned EDOT oligomeric species suspended in the aqueous phase are not likely to be involved in the formation of the PEDOT thin film at the L|L interface, as discussed in the Supporting Information.

Potentiostatic experiments holding  at +0.4 V for 1000 s were also studied (Figure S13). In this case, the quantity of cationic EDOT oligomer adsorption was minimal due to aqueous SO_4_^2–^ anions being unavailable to undergo ion-pairing at +0.4 V at the L|L interface. Furthermore, oligomer adsorption is a balance between the interfacial tension (γ) and the oligomer size. Therefore, factors that lower γ , such as applied potentials close to the positive or negative ends of the polarizable potential window,^43^ may slow the adsorption kinetics as the oligomers’ “critical size” increases. Nevertheless, after 1000 s, a PEDOT thin film could be seen coating the L|L interface. However, this thin film was not mechanically robust enough to recover for ex situ characterization in comparison to films prepared by DPSCA.

### Microscopic Analysis

**Figure 3:**
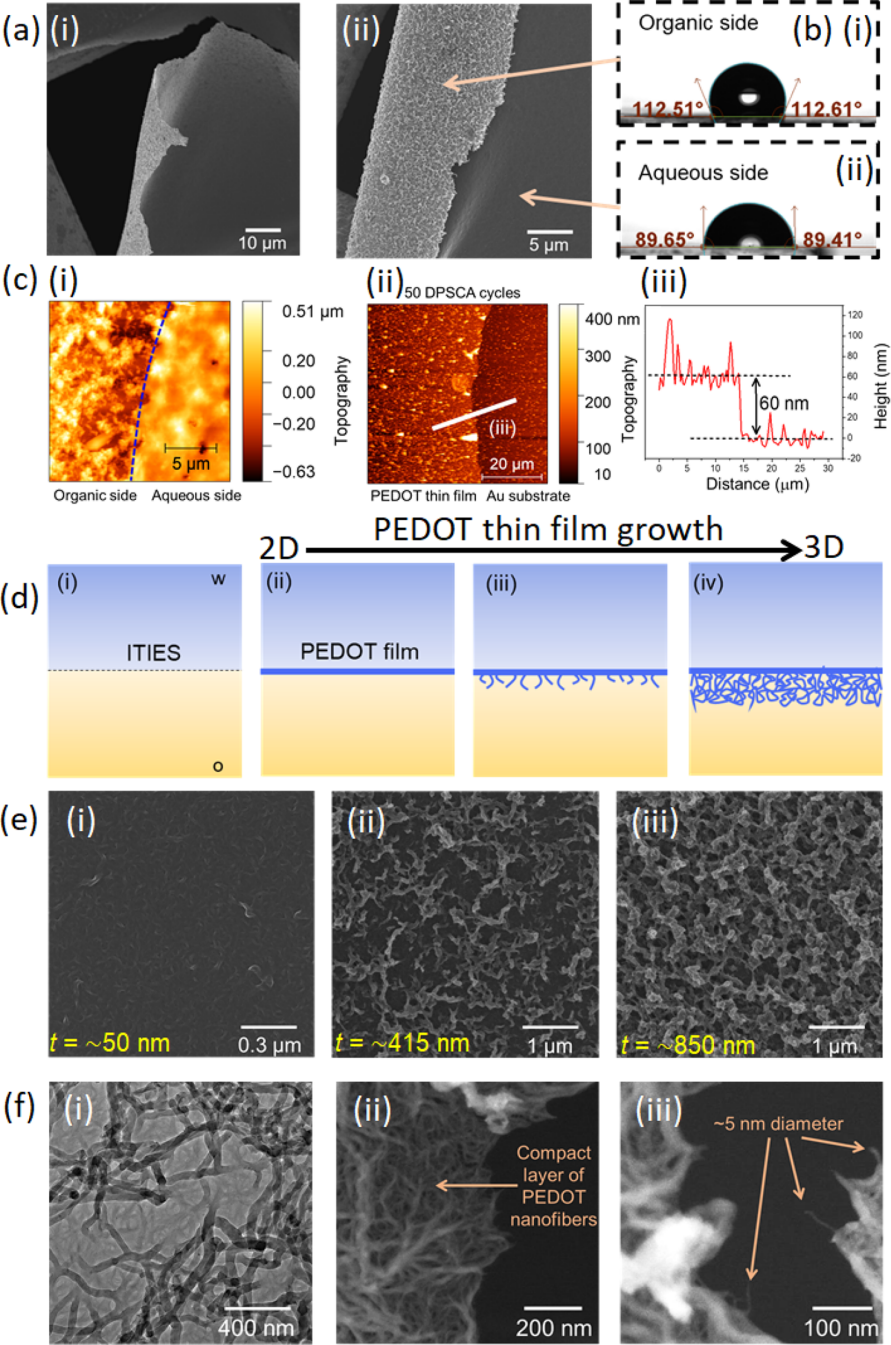
Microscopic analysis of the PEDOT thin film. (a) Scanning electron microscopy (SEM) images of a free-standing 2D PEDOT film electrosynthesized at the ITIES, showing the distinct morphologies of the “smooth” aqueous-facing side and “rough” organic-facing side. Additional SEM images are provided in the Supporting Information. (b) Sessile drop contact angle measurements recorded on each side of the PEDOT thin film, highlighting the influence of the morphology on the measured hydrophobicity. (c) Atomic force microscopy (AFM) analysis of (i) the topography of a PEDOT thin film folded back on itself and (ii, iii) the thickness of a PEDOT thin film on a flat gold substrate after 50 DPSCA cycles. (d) Schematic of the different stages of PEDOT thin film growth from 2D to 3D as a function of continued DPSCA cycling. (e) SEM images showing the organic-facing side of the PEDOT thin film, demonstrating the controllable growth on the organic-facing side of the thin film from 2D to 3D as a function of DPSCA cycling. The thickness (*t*) of each thin film was determined by AFM (Figure S17). (f) (i) Bright-field and (ii, iii) dark-field mode transmission electron microscopy (TEM) imaging shows that the film is a compact network of PEDOT nanofibers that have diameters that range from <5 nm up to 50 nm. The arrows in (iii) point to PEDOT nanofibers with a diameter of ca. 5 nm. All PEDOT thin films analyzed were prepared by DPSCA cycling using the cell configuration outlined in Figure 2a.

Scanning electron microscopy (SEM) of a 2D PEDOT thin film prepared by DPSCA (150 cycles) revealed an asymmetric “Janus” morphology (see Figure 3a, Supporting Information, and Figure S14). One side is flat and featureless at the nanoscale, while the other shows a rough, porous 3D structure. The PEDOT thin films adhere to any solid substrate, with the thin film closely following the contours and taking the shape of the surface (Figures S15 and S16). The asymmetric nature of the PEDOT thin film leads to each side having distinct physical properties. For example, sessile drop measurements highlight significant differences in hydrophobicity, with water droplet contact angles of 89.5 and 112.6° measured on the flat and rough sides, respectively (Figure 3b). Atomic force microscopy (AFM) images of the topography of a PEDOT thin film folded back on itself clearly highlight the differences in surface roughness of either side (Figure 3ci) and revealed that the thickness of a thin film prepared by 50 DPSCA cycles is ca. 60 nm (Figure 3cii,iii).

The evolution of the morphology of the organic-facing side of the PEDOT thin film with DPSCA cycling is depicted schematically in Figure 3di–iv, with SEM images of each stage provided (Figure 3ei–iii) and the thickness of each stage determined by AFM (Figure S17). Initially, after 50 DPSCA cycles, the thin film shows 2D growth parallel to the ITIES, a highly compact structure that is flat on both sides, and a thickness of 40–60 nm (Figure 3ei). With continued interfacial electrosynthesis (up to 150 DPSCA cycles), secondary 3D growth begins to extend into the organic phase as the thickness of the PEDOT thin film increases (Figure 3eii). This controllable secondary growth process leads to the formation of a very porous 3D structure with a thickness of up to ∼850 nm after prolonged (>300 DPSCA cycles) interfacial electrosynthesis (Figure 3eiii).

Transmission electron microscopy (TEM) studies revealed that the PEDOT thin film is exceptionally stable under the TEM beam (80 kV), signaling a high thermal conductivity and providing an opportunity to further investigate the PEDOT thin film’s nanostructure. Both bright-field (Figure 3fi) and dark-field (Figure 3fii–iii) mode TEM images show that the flat aqueous side consists of a compact layer of PEDOT nanofibers that run parallel to the ITIES. The diameter of the PEDOT nanofibers varies from <5 nm to above 50 nm. We propose that the nanofibers with a diameter of <5 nm are first to be deposited at the ITIES during interfacial electrosynthesis, forming an initial compact layer. Subsequently, the nanofiber diameter increases as the thin film grows down into the organic phase.

### Spectroscopic, Conductivity, and Electrochemical Analysis

Ex situ spectroscopic analysis was performed on PEDOT thin films transferred to suitable solid substrates (Supporting Information). A bipolaron band was observed by UV–Vis absorbance, signifying that the PEDOT thin film is in an oxidized state^44^ and *p*-doped (Figure S18). Raman spectroscopy also confirmed that the PEDOT thin film is *p*-doped, with high π–π conjugation and a benzenoid (coiled) configuration to the polymer chain (Figure S19), the more stable form when PEDOT is highly doped.^45−47^ An X-ray-photoelectron spectroscopy (XPS) survey spectrum showed the presence of only sulfur, carbon, and oxygen (Figure S20), indicating that the cerium oxidant, boron, and fluorine are not incorporated into the thin film. This implies that the organic TB^–^ anion is not involved in *p*-doping and SO_4_^2–^ is the primary dopant. Further XPS analysis revealed that the doping level was estimated as ∼39% (Figure S21), in line with previous reports of the upper limits possible of PEDOT doping between 35–40%.^48,49^

Excess negative charge from the insulating surfactant PSS can detrimentally alter the conduction mechanism of CP thin films;^50^ for more details, see the Supporting Information*.* Ex situ conductivity of the PEDOT thin film was determined as 554 (±77) S·cm^–1^ (Supporting Information and Figure S22), comparable to the highest conductivity value reported for a pristine PEDOT:ClO_4_^–^ film (400–650 S·cm^–1^) made by conventional electropolymerization at a solid electrode surface in acetonitrile.^51,52^ The in situ conductivity of a PEDOT thin film and a commercial PEDOT:PSS film drop-cast and annealed directly onto a microelectrode array were compared in PBS buffer solution (Figure S23). The PEDOT and PEDOT:PSS films had maximum conductivities of ∼5.35 and ∼1.2 S·cm^–1^, respectively, in their plateau regions, with the conductance window in the PEDOT thin film 0.4 V greater than that of the PEDOT:PSS film (Figure S24). The latter is advantageous for OECT devices working at lower potentials to avoid oxidative reactions or biological stress (if the active layer is to be functionalized with cells). Corresponding CVs of each film in PBS buffer solution were obtained using a range of scan rates and potential ranges (Figures S25 and S26). The PEDOT thin film remains doped over a very wide potential range (in comparison to the PEDOT:PSS film) and therefore has high conductivity even at negative potentials. At high scan rates, the PEDOT thin film displays an ideal capacitive behavior due to the polymer films’ high intrinsic conductivity and wide conductance window.

### Biocompatibility Studies

**Figure 4:**
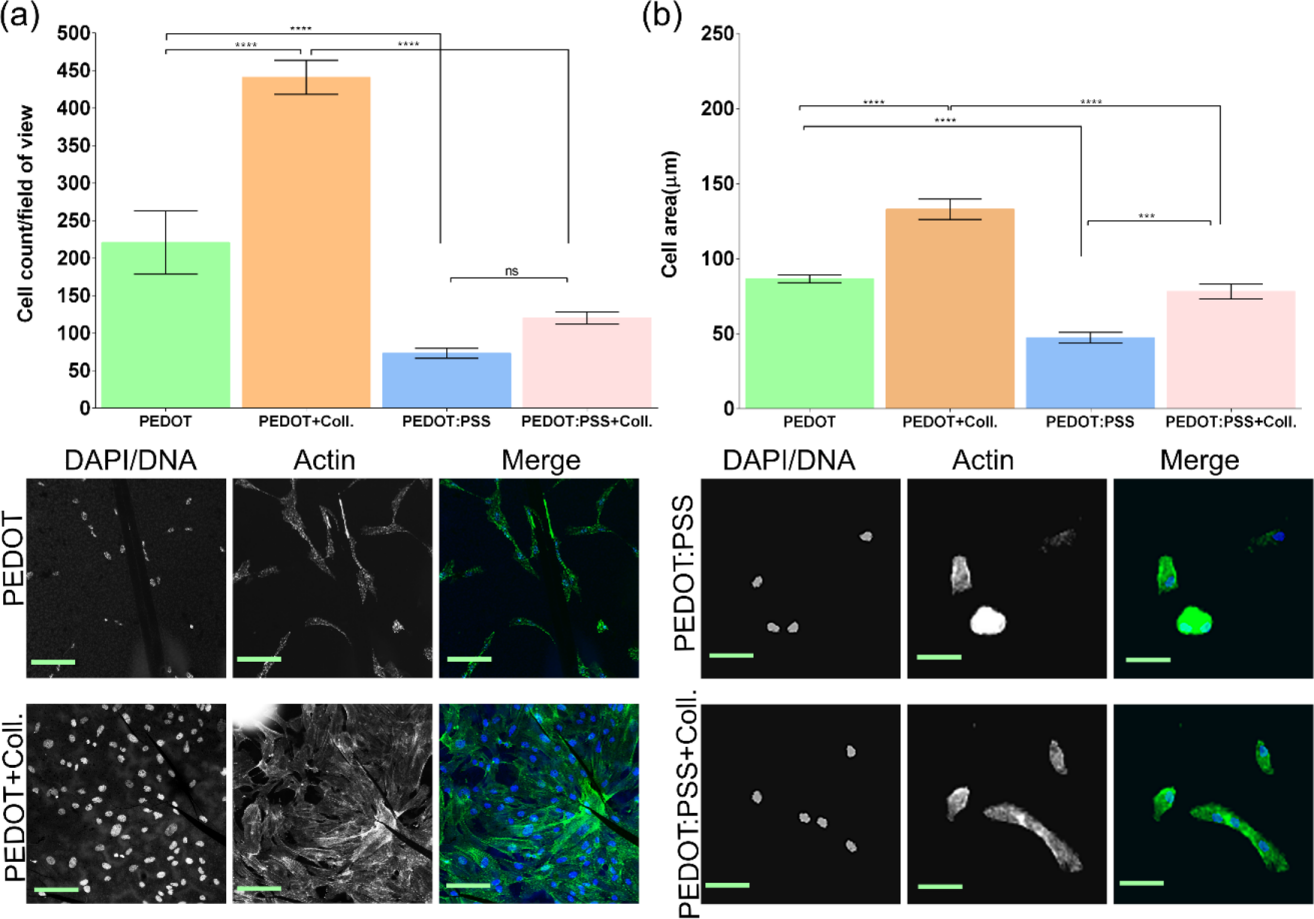
Biocompatibility of PEDOT thin films prepared at the ITIES and drop-cast PEDOT:PSS films with and without collagen functionalization. (a) Cell proliferation analysis. (b) Cell area analysis for each sample film. Scale bar, 50 μm.

PEDOT:PSS films are being extensively explored for the treatment of visual impairment, particularly relating to amelioration or restoration of retinal function.^30,31^ However, the action of PSS severely hampers the biocompatibility and integrity of the resultant implantable biodevices (Supporting Information). In this context, the biocompatibility of PEDOT thin films electrosynthesized at the ITIES was compared with that of a drop-cast PEDOT:PSS film using an adherent cell line derived from normal human retina pigment epithelium (hTERT RPE-1) (see Figure 4).

Following 48 h of cell growth on each film, marked differences were observed in the hTERT RPE-1 cell growth dynamics (Figure 4). Overall, in the presence of PEDOT thin films, cells exhibited a greater degree of proliferation and showed a stretched morphology associated with actin bundle stress fiber formation, alluding to the more biocompatible nature of PEDOT versus PEDOT:PSS (Figure 4a,b). These data reinforce the challenges of using PSS at biointerfaces and differentiate our investigations from many evaluating PEDOT:PSS in cell culture, where the lack of obvious cytotoxicity is incorrectly construed as high biocompatibility. Next, each film was evaluated for the suitability of active biomolecule incorporation. Collagen was selected as an active biomolecule due to its known influence on cell proliferation and adhesion, which could potentially mitigate the poor cellular growth seen on PEDOT:PSS samples. While only marginal effects were observed in cells grown on PEDOT:PSS films, the functionalization of PEDOT thin films resulted in a marked amplification in cell proliferation and cell spreading, indicative of robust adhesion receptor engagement and bioactivation of the cell cycle program (Figure 4a).

## Conclusions

Interfacial electrosynthesis bridges the fields of purely homogeneous (chemical) electron transfer reactions between redox couples, which are difficult to control, and finely controlled (electrochemical) heterogeneous electron transfer at conventional solid electrode–electrolyte interfaces. Polarizing the L|L interface provides a built-in ability to control the kinetics of IET between oxidant and monomer redox couples in opposite phases and probe the mechanism in situ electrochemically. These two features elevate this work over studies that involve spontaneous interfacial reactions (polycondensation, polyaddition, and self-assembly),^53−56^ which permit only rudimentary chemical control over reaction kinetics and a limited ability to study the synthetic mechanism in situ. Our work unravels the underlying thermodynamic limitations and mechanism of PEDOT thin film interfacial electrosynthesis that progresses along five distinct stages with time. This underlying mechanism will be broadly generic for all aqueous oxidant/organic monomer combinations that are thermodynamically compatible to facilitate IET within the Galvani polarizable potential window (where the IET kinetics are under direct external electrochemical control). Such an understanding will allow us to identify suitable oxidant/monomer combinations, opening the field of interfacial electrosynthesis to other technologically critical CPs beyond PEDOT, e.g., poly(3-hexylthiophene-2,5-diyl), commonly known as P3HT.

The PEDOT thin films prepared by interfacial electrosynthesis showed superior biocompatibility and suitability to be used as a scaffold for cellular growth without the need for further modification designed to promote cell adhesion or enhance viability. Bioactive molecules can be readily incorporated into PEDOT polymers as a further customizable parameter for cell growth studies. This opens an attractive avenue for potential new and improved OECT devices for monitoring cell behavior over extended time periods, bioscaffolds, and medical devices without the requirement for physiologically unstable and poorly biocompatible PSS. The dimensions and geometry of free-standing thin films made possible by interfacial electrosynthesis are only limited by the interfacial area and shape defined by the electrochemical cell. Furthermore, we demonstrate that interfacial electrosynthesis is a local and mild process that requires minimal reagents (e.g., low oxidant concentrations) and is thus highly compatible with large-scale thin film production. In this regard, future work will explore polarizing the L|L interface chemically by establishing a suitable distribution potential through ion partition between the two phases to scale up thin film production.

## Supporting Information Available

Supplementary materials and methods; mechanism and thermodynamics of PEDOT interfacial electrosynthesis; electrochemical initiation, control, and monitoring of PEDOT interfacial electrosynthesis; microscopic (SEM, TEM), spectroscopic (UV–Vis, Raman, and XPS), conductivity, and electrochemical properties of the PEDOT thin films; biocompatibility studies using PEDOT thin films (PDF)
