# Supplementary material for: Electrosynthesis of Biocompatible Free-Standing PEDOT Thin Films at a Polarized Liquid|Liquid Interface
Source: J Am Chem Soc. 2022 Mar 9;144(11):4853–62. doi: 10.1021/jacs.1c12373 (PMC8949726; doi:10.1021/jacs.1c12373)
Supplement: Supplementary file 1 — ja1c12373_si_001.pdf [file ja1c12373_si_001.pdf]

# Supporting Information

## Electrosynthesis of Biocompatible Free-Standing PEDOT Thin Films at a Polarised Liquid|Liquid Interface

Rob A. Lehane,[a,b,†] Alonso Gamero-Quijano,*[,a,b,†] Sigita Malijauskaite,[a,b] Angelika Holzinger,[a,b] Michele Conroy,[a,c] Fathima Laffir,[a] Amit Kumar,[d] Ursel Bangert,[a,c] Kieran McGourty,[a,b,e] and Micheál D. Scanlon*[,a,b,f]

[a] Bernal Institute, University of Limerick (UL), Limerick V94 T9PX, Ireland

[b] Department of Chemical Sciences, School of Natural Sciences, University of Limerick (UL), Limerick V94 T9PX, Ireland

[c] Department of Physics, School of Natural Sciences, University of Limerick (UL), Limerick V94 T9PX, Ireland

[d] School of Mathematics and Physics, Queen's University Belfast (QUB), Belfast BT71 NN, UK

[e] Health Research Institute (HRI), University of Limerick (UL), Limerick V94 T9PX, Ireland

[f] The Advanced Materials and Bioengineering Research (AMBER) centre, CRANN Institute, Trinity College Dublin (TCD), Dublin 2, D02 PN40, Ireland

[†] These authors contributed equally to the work.

* Corresponding author: daniel.gamero@ua.es

* Corresponding author: micheal.scanlon@ul.ie

**Table of Contents**


| | |
|---|---|
| **Section S1: Supplementary material and methods** | S3 |
| **Section S2. The mechanism of PEDOT interfacial electrosynthesis** | S6 |
| **Section S3. The thermodynamics of PEDOT interfacial electrosynthesis** | S10 |
| **Section S4. Electrochemically initiating, controlling, and monitoring PEDOT thin film interfacial electrosynthesis** | S15 |
| 4.1 Trends in the kinetics of interfacial electrosynthesis with double potential step chronoamperometry (DPSCA) cycle number | S15 |
| 4.2 Interfacial ion-pairing and interchange between adsorbed cationic EDOT oligomers and aqueous $SO_4^{2-}$ anions | S17 |
| 4.3 *In situ* parallel beam UV/vis absorbance measurements | S18 |
| 4.4 Using the Tyndall effect to explore the partition of small EDOT oligomers | S19 |
| 4.5 Potentiostatic polarisation of the L\|L interface | S21 |
| **Section S5. Microscopic analysis of the PEDOT thin film** | S22 |
| 5.1 Scanning electron microscopy (SEM) analysis | S22 |
| 5.2 Atomic force microscopy (AFM) analysis | S26 |
| **Section S6. Spectroscopic analysis of the PEDOT thin films** | S27 |
| 6.1 UV/vis-NIR absorbance | S27 |
| 6.2 Raman spectroscopy | S28 |
| 6.3 X-ray photoelectron spectroscopy (XPS) | S29 |
| **Section S7. Conductivity and electrochemical properties of the PEDOT thin film** | S32 |
| 7.1 The impact of excess PSS on conductivity, long-term stability and specific capacity | S32 |
| 7.2 *Ex situ* and *in situ* conductivity of the PEDOT thin film | S32 |
| 7.3 Electrochemical characterisation of the PEDOT thin film in a 3-electrode configuration immobilised on a solid electrode surface | S37 |
| **Section S8. Biocompatibility studies using PEDOT thin films** | S41 |
| 8.1 Biocompatibility studies: detailed sample preparation | S41 |
| 8.2 PSS severely hampers the biocompatibility and integrity of implantable biodevices | S42 |
| **Supplementary references** | S44 |

## Section S1: Supplementary materials and methods

**Materials.** All chemicals were used as received without further purification. All aqueous solutions were prepared with ultra-pure water (Millipore Milli-Q, specific resistivity 18.2 MΩ·cm). The organic solvent α,α,α-trifluorotoluene (TFT, 99+%) was received from Acros Organics. The organic monomer 3,4-ethylenedioxythiophene (EDOT, 97%), cerium (IV) sulfate ($Ce(SO_4)_2$, 99%), sulfuric acid ($H_2SO_4$, 95.0%), lithium chloride (LiCl, ≥99%) and tetraethylammonium chloride (TEACl, 99%) were purchased from Sigma-Aldrich. Lithium tetrakis(pentafluorophenyl)borate diethyletherate (LiTB) was received from Boulder Scientific Company. The organic electrolyte salt bis(triphenylphosphoranylidene)ammonium tetrakis(pentafluorophenyl)borate (BATB) was prepared by metathesis of equimolar solutions of BACl and LiTB in a methanol-water (2:1 v/v) mixture. The resulting precipitates were filtered, washed and recrystallised from acetone. For comparative studies, a PEDOT:PSS ink (1.1% in $H_2O$, surfactant-free, high-conductivity grade) was purchased from Sigma-Aldrich.

For biocompatibility studies, Dulbecco's Modified Eagle's Medium (DMEM), 10% fetal bovine serum (FBS), 1% penicillin/streptomycin, sodium bicarbonate (S8761), collagen in phosphate buffer saline (PBS) solution (125-50) Trypsin-EDTA solution (T4049), anti-actin FITCE conjugated dye (19083) and 4′,6-diamidino-2-phenylindole (DAPI) were obtained from Sigma-Aldrich. Also, 1% GlutaMAX (35050061) was obtained from ThermoFisher Scientific, Zombie green (423111) from Biolegend and normal human Retina Pigment Epithelium (hTERT RPE-1; CRL-4000) from ATCC.

**Interfacial electrosynthesis at a polarised aqueous|organic interface.** Interfacial electrosynthesis was carried out at an ITIES formed between an acidic aqueous solution, containing 0.2 M $H_2SO_4$ electrolyte and 2 mM $Ce(SO_4)_2$ as the oxidant, and an organic TFT solution, containing 5 mM BATB electrolyte and 5 mM EDOT as the monomer (see Figure

2a). The interfacial Galvani potential difference at the polarised aqueous|TFT interface ($\Delta_{o}^{w}\phi$) was controlled externally using an Autolab PGSTAT204 from Metrohm (Netherlands), in conjunction with NOVA software version 2.1.2. and a specialised four-electrode electrochemical cell. A detailed schematic of the latter is shown in Figure S4 and images are provided in Figure 1b. A theoretical background to such experiments at an ITIES can be found in several articles and book chapters.[1–5] All electrochemical measurements were carried out in ambient aerobic conditions at room temperature (RT, 21$^{o}$C). The methodology to calibrate all electrochemical data obtained at the ITIES to the Galvani potential scale is described in Section S2.

Interfacial electrosynthesis was initiated using a double potential step chronoamperometry (DPSCA) method. The first potential step was held at $\Delta_{o}^{w}\phi$ = +0.4 V for 10 s. The second potential step was then held at $\Delta_{o}^{w}\phi$ = –0.1 V for 10 s. This double potential step was repeated up to 300 times, depending on the desired thickness of the PEDOT thin film. AC voltammetry was performed after each of the first five DPSCA cycles. as shown in Figure 2c. Differential capacitance was calculated from the interfacial admittance recorded using an Autolab FRA32M module in combination with the Autolab PGSTAT204 at a frequency of 10 Hz and root mean square (RMS) amplitude of 10 mV. The scan direction was from negative towards more positive potentials, from ca. –0.20 to +0.35 V.

**Biocompatibility studies.** An adherent cell line of normal human Retina Pigment Epithelium (hTERT RPE-1) of passage number <10 was used for this study. Details of the methods to seed and culture these cells on PEDOT thin films prepared by interfacial electrosynthesis and PEDOT:PSS films (for control experiments), with and without collagen present, are provided in Section S8.1.

For the biocompatibility study, samples were imaged on the ImageXpress Micro Confocal High-Content Imaging System (Molecular Devices) with a 10X S Plan Apo objective.

9 fields of view per sample in each wavelength were analysed using open source CellProfiler software (v 4.0.5).[6] Briefly, image sets corresponding to various fluorescent channels in each field of view were subjected to normalization and analysis pipelines where cell object segmentation was undertaken using DAPI nuclear identification. Mean cell areas were quantified using ImageJ, by the bounding pixel area associated with segmented objects denoted through actin labelling.

Statistical analysis was performed using Graph Pad Prism Version 8.4.2 (464) (GraphPad Software, La Jolla, CA, USA), and values tested for significance using one-way ANOVA followed by Tukey post-hoc tests, or t-tests for pair-wise comparison. Statistical tests were two tailed with a significance level of $\alpha \leq 0.05$. Significances are stated with p values < 0.05 *; <0.01 **; <0.001 ***. Results are shown as standard error of means.

## Section S2. The mechanism of PEDOT interfacial electrosynthesis

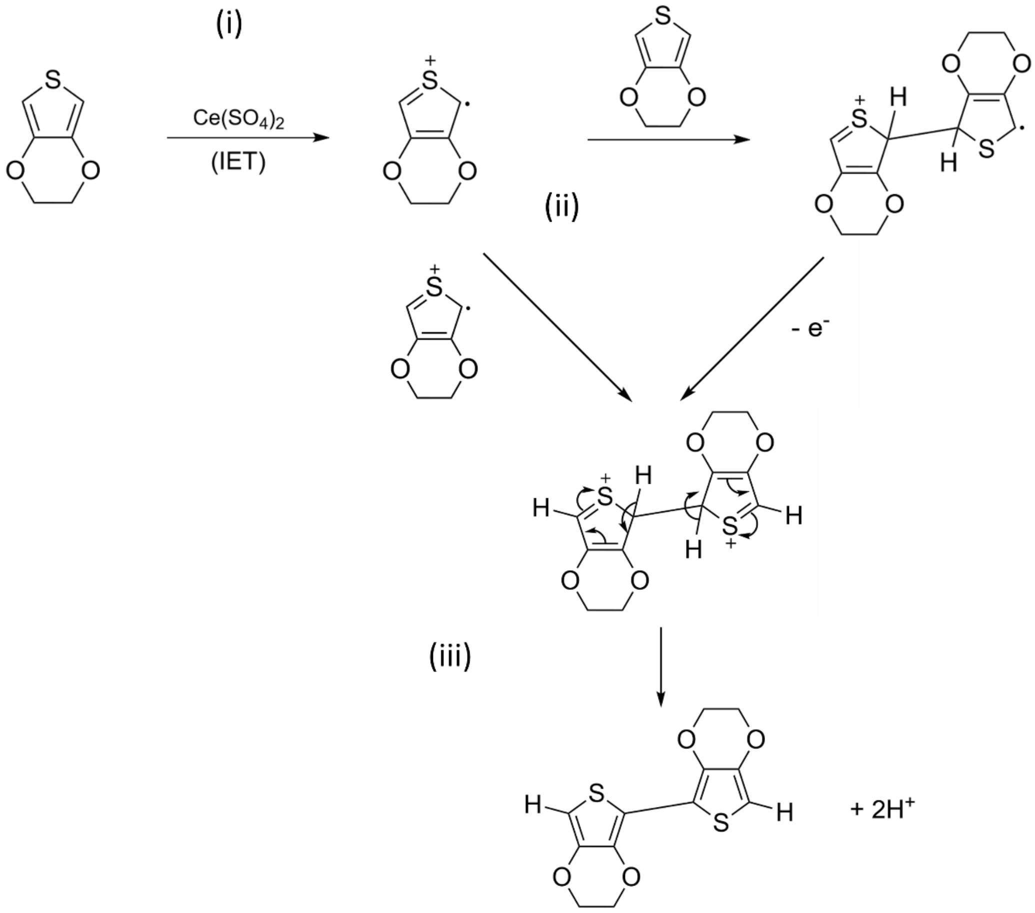


**Figure S1. Proposed mechanism of interfacial PEDOT thin film formation: the interfacial electron transfer (IET)/radical coupling and interfacial adsorption steps (see Figure 1(a)(i) and (ii)).** (i) Organic EDOT is oxidised by aqueous $Ce^{4+}$ to a radical cation ($EDOT^{\bullet+}$) at the polarised L|L interface; (ii) $EDOT^{\bullet+}$ species couple with each other or another EDOT monomer to form dimers that (iii) subsequently get deprotonated. Electroneutrality is maintained in the diffusion zone on the organic side of the interface by electrostatic interactions between the cationic oligomers and organic tetrakis(pentafluorophenyl)borate anions ($TB^{-}$).

The removal of protons to the aqueous phase is essential to maintain electroneutrality in both the aqueous and organic phases and ensure IET from EDOT to $Ce^{4+}$ is not electrostatically

inhibited. On the organic side of the ITIES an accumulation of positive species ($H^+$, $EDOT^{\bullet+}$, cationic oligomers) is compensated by the transfer of protons to the aqueous. Furthermore, as $Ce^{4+}$ is reduced to $Ce^{3+}$, protons pumped to the aqueous side of the ITIES will maintain electroneutrality there.

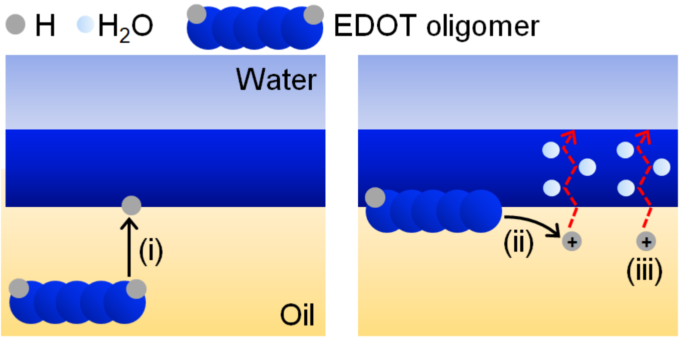


**Figure S2. Schematic showing the release of protons in the organic phase during PEDOT interfacial electrosynthesis.** (i) EDOT oligomers in the organic phase react with the existing PEDOT thin film at the aqueous|TFT interface *via* a radical-radical coupling mechanism, (ii) with protons being released. (iii) Protons transfer through the film to the aqueous phase by Grotthuss diffusion.

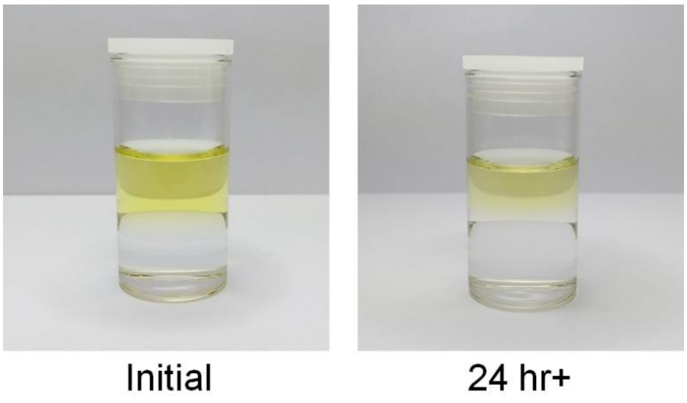


**Figure S3. The need to polarise the L|L interface to initiate and control interfacial electropolymerisation of PEDOT thin films.** Upon contact of an aqueous solution of 2 mM $Ce^{4+}$ in 0.2 M $H_2SO_4$ (top phase) with an organic solution of 5 mM EDOT and 5 mM BATB

in TFT (bottom phase), no PEDOT thin film forms after more than 24 hr. Interfacial electron transfer (IET) may occur at a very slow kinetic rate at open circuit potential (OCP) conditions, as evidenced by the loss in yellow colouration due to reduction of yellow $Ce^{4+}$ to colourless $Ce^{3+}$ in the diffusion layer directly above the L|L interface. Nevertheless, no PEDOT thin film forms.

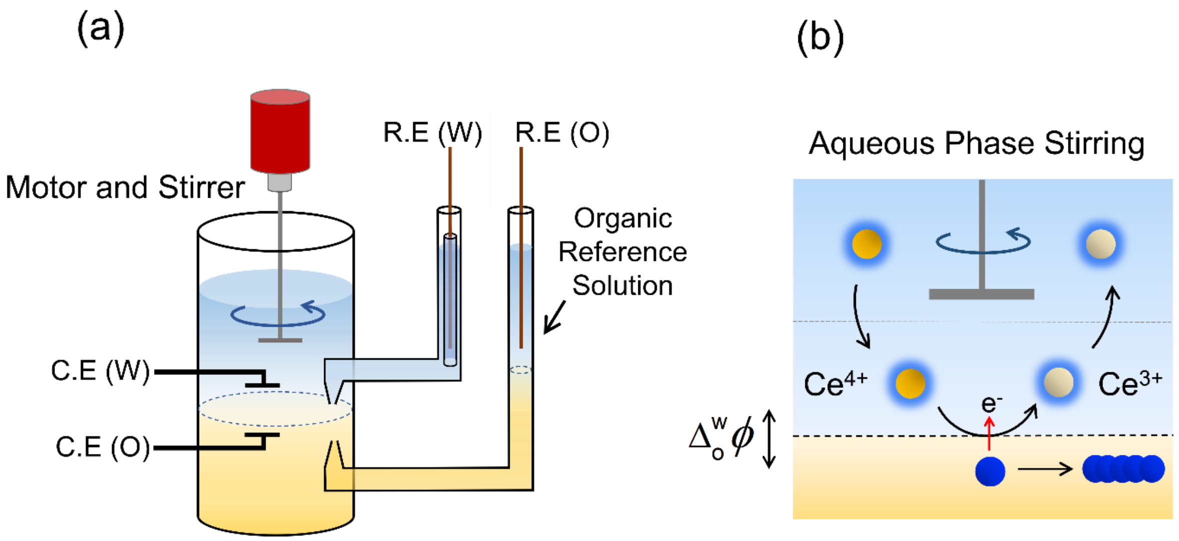


**Figure S4**. **Schematic of the four-electrode electrochemical cell used to polarise the L|L interface.** (a) Using Luggin capillaries, two Ag/AgCl reference electrodes (R.E) were positioned on either side of the aqueous|TFT interface to measure the potential difference across the interface. Platinum counter electrodes (C.E.) were placed in each phase to allow the flow of electric current. The geometrical surface area of the aqueous|TFT interface was ~1.66 $cm^2$. (b) To enhance the diffusion of the $Ce^{4+}$ oxidant in the aqueous phase, a homemade stirrer was positioned approx. 2 cm above the aqueous|TFT interface. A glass capillary with a U-bend was used as the stirrer, which was attached to an electric motor that operated at 240 rpm. The chemical compositions of the aqueous and organic phases to achieve free-standing PEDOT thin film formation are described in Figure 2a in the main text.

To calibrate the electrochemical measurements obtained to the Galvani potential scale, the applied potential ($E$) in the four-electrode cell at the polarised aqueous|TFT interface is defined as the potential difference established between the Ag/AgCl reference electrode in the aqueous phase and that in the organic reference solution. The applied potential ($E$) encompasses the interfacial Galvani potential difference ($\Delta_{\mathrm{o}}^{\mathrm{w}}\phi$). The latter is defined as $\Delta_{\mathrm{o}}^{\mathrm{w}}\phi = (\phi^{\mathrm{w}} - \phi^{\mathrm{o}})$, where $\phi^{\mathrm{w}}$ and $\phi^{\mathrm{o}}$ are the inner Galvani potentials of the aqueous and organic phases, respectively. Additionally, the applied potential ($E$) is determined by the nature of the reference electrodes used. These contributions to the applied potential ($E$) are defined here as $\Delta E_{\mathrm{ref.}}$. The calibration of the cyclic voltammograms obtained at the polarised aqueous|TFT interface to the Galvani potential scale was performed following the relationship $E = \Delta_{\mathrm{o}}^{\mathrm{w}}\phi + \Delta E_{\mathrm{ref.}}$. The critical value of $\Delta E_{\mathrm{ref.}}$ was determined using the electrochemical half-wave ion transfer response of tetraethylammonium cations ($TEA^+$; $E_{1/2,\mathrm{TEA}^+}^{\mathrm{w}\rightarrow\mathrm{TFT}}$) and the standard ion transfer potential of $TEA^+$ from the aqueous to TFT phase (known to be $\Delta_{\mathrm{o}}^{\mathrm{w}}\phi_{\mathrm{tr.,TEA}^+}^{\ominus,\mathrm{w}\rightarrow\mathrm{TFT}} = 0.149$ V).[7]

## Section S3. The thermodynamics of PEDOT interfacial electrosynthesis

A brief discussion of the underlying thermodynamics for this biphasic system explains the need for an external electrochemical driving force. The standard Galvani potential for IET ($\Delta_{o}^{w}\phi_{IET}^{\ominus}$) represents the Galvani potential ($\Delta_{o}^{w}\phi$) where the interfacial redox reaction is at equilibrium. This equilibrium potential is assumed to be the half-wave potential of the reversible redox process ($\Delta_{o}^{w}\phi_{1/2}$) and may be determined from the standard redox potentials of the aqueous (w) and organic (o) redox species, respectively, both expressed *vs.* the standard hydrogen electrode (SHE), using Equation (S1):

$$\Delta_{o}^{w}\phi_{IET}^{\ominus} = \Delta_{o}^{w}\phi_{1/2} = \left(\left[E_{Ox/Red}^{\ominus}\right]_{SHE}^{o} - \left[E_{Ox/Red}^{\ominus}\right]_{SHE}^{w}\right) \qquad (S1)$$

Using a polycrystalline gold electrode in a three-electrode configuration, the redox potential of the aqueous $Ce^{4+}$ oxidant in 0.2 M $H_2SO_4$ was determined as +1.430 V *vs.* SHE (Figure S5), in agreement with literature values.[8] Meanwhile, the redox potential of EDOT was estimated from the onset potential of EDOT oxidation in the organic TFT solvent (not reported in the literature to date) at a polycrystalline platinum electrode as +1.435 V *vs.* SHE (Figure S6). Thus, from Equation (S1), $\Delta_{o}^{w}\phi_{IET}^{\ominus}$ is ca. 0 V and IET can only proceed with appreciable kinetics by applying an external driving force.

A closed bipolar electrochemical cell (CBPEC) in a four-electrode configuration was also employed to directly determine $\Delta_{o}^{w}\phi_{1/2}$ for biphasic PEDOT electrosynthesis on the Galvani scale (see Figure S7 for a detailed description of this biphasic methodology). IET between redox couples in a CBPEC configuration is thermodynamically equivalent to the corresponding event at the ITIES.[9–11] Using the CBPEC, $\Delta_{o}^{w}\phi_{IET}^{\ominus}$ for biphasic PEDOT electrosynthesis was confirmed as ca. 0 V (see Scheme S1 and Figure S8). Compared with the CPBEC experiments, additional overpotentials ($\eta$) to IET across the ITIES, such as

reorganisation energies and double-layer effects,[9–11] will push $\Delta_o^w\phi$ for IET more positive ($\Delta_o^w\phi = \Delta_o^w\phi_{1/2} + \eta$). The maximum Galvani polarisable potential window (PPW) ranges from –0.4 to +0.6 V at an aqueous|TFT interface.[12] With expected overpotentials of ca. 200 mV, IET between $Ce^{4+}$ and EDOT is predicted to occur at positive potentials >+0.2 V. Therefore, as the latter lies within the limits of the available Galvani PPW, the kinetics of PEDOT interfacial electrosynthesis are directly under external electrochemical control.

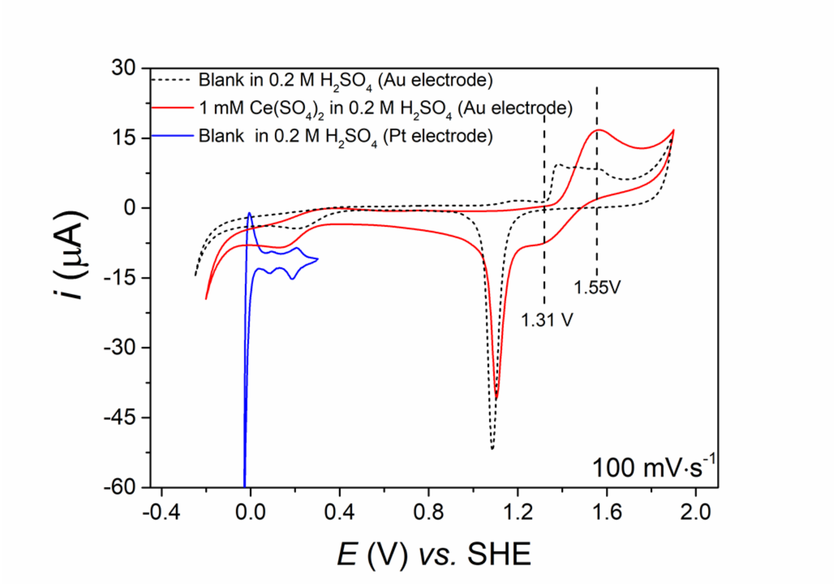


**Figure S5. The redox potential of $Ce^{4+}/Ce^{3+}$ in 0.2 M $H_2SO_4$ was determined by cyclic voltammetry (CV) using a 3-electrode electrochemical cell.** The redox potential for $Ce^{4+}/Ce^{3+}$ in 0.2 M $H_2SO_4$ was measured at 1.43 V (*vs.* SHE) which is in agreement with the literature values.[8] The working electrode was a polished Au disc electrode (CH instruments, USA), the counter electrode was a Pt wire, and the reference was a Ag/AgCl(KCl gel) electrode (Pine Instruments, USA). The surface of the Au working electrode was cleaned electrochemically in 0.2 M $H_2SO_4$ by CV prior to the addition of 1 mM $Ce(SO_4)_2$. The measured potential was converted to the standard hydrogen potential (SHE) scale using a polycrystalline Pt working electrode, where the onset of HER takes place at 0 V (*vs.* SHE).[13] CVs were obtained at a scan rate of 20 $mV\cdot s^{-1}$.

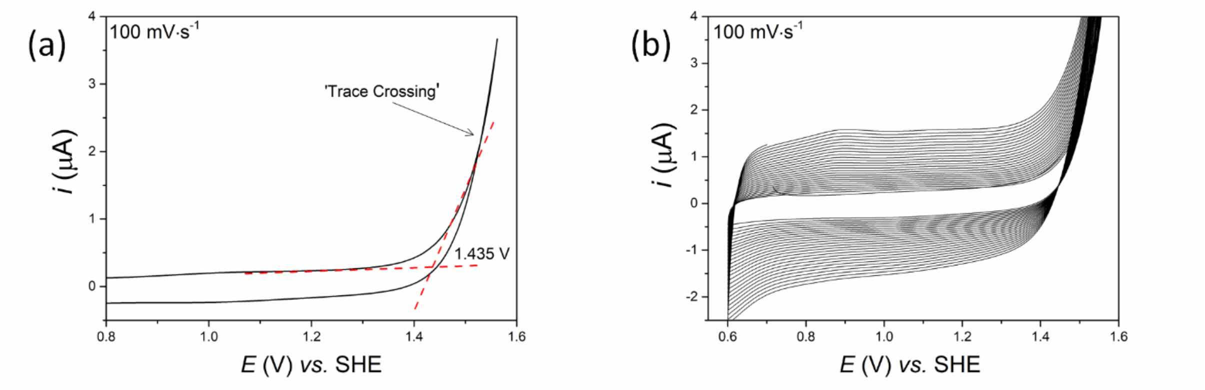


**Figure S6. The onset of EDOT oxidation in an organic electrolyte solution of 5 mM BATB in TFT was determined by CV using a modified 3-electrode electrochemical cell**.[14] (a) The redox potential of EDOT was estimated from the onset of EDOT oxidation in TFT was measured at 1.435 V (*vs.* SHE). (b) The magnitude of the charge on the forward and reverse sweeps increased with each CV cycle as the PEDOT film grew in thickness on the working electrode surface. The working electrode was a polished Au disc electrode (CH instruments, USA) and the counter electrode was a Pt wire. The reference electrode was a Ag/AgCl wire, which was immersed in an 'organic reference solution' that established a liquid junction with the organic solvent. The organic reference solution was an aqueous solution of 1 mM LiCl and 10 mM BACl. Before use, the Au working electrode was cleaned electrochemically in 0.2 M $H_2SO_4$ and washed with DI water, acetone and TFT solvent. The measured potential was calibrated to the SHE potential scale using the decamethyferrocene ($DcMFc^+$ /DcMFc) redox couple in TFT, which has a standard redox potential of 0.107 V (*vs.* SHE).[14] CVs were obtained at a scan rate of 100 mV·$s^{-1}$.

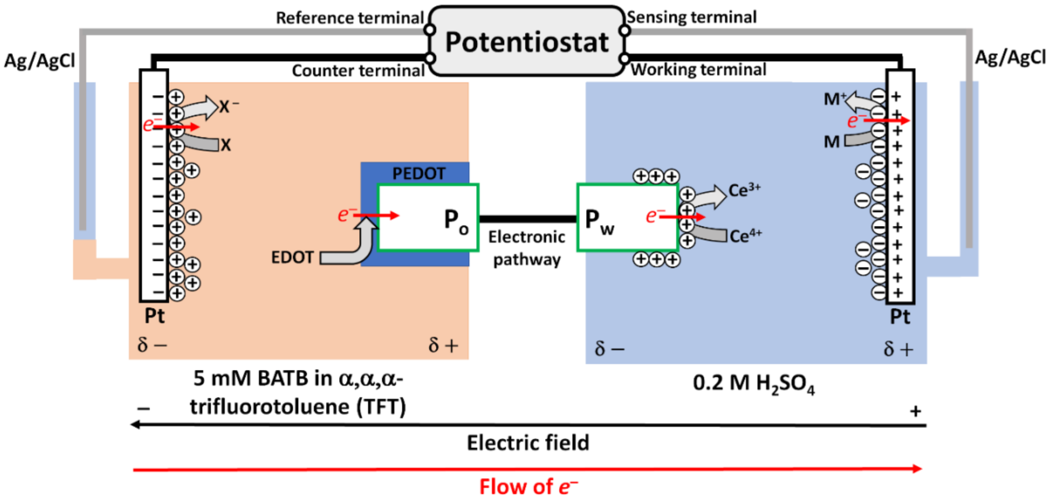


**Figure S7. Detailed schematic of the four-electrode closed bipolar electrochemical cell (CBPEC) configuration with immiscible aqueous|organic electrolyte solutions for the electropolymerisation of organic EDOT by aqueous $Ce^{4+}$**. As the Galvani potential difference between the aqueous and organic phases ($\Delta_o^w\phi$) is biased positively by the potentiostat, this facilitates electron transfer along the bipolar electrode (BPE) from the EDOT, which is oxidised forming a PEDOT film at the organic pole ($P_o$), to aqueous $Ce^{4+}$, which is reduced at the aqueous pole ($P_w$). The schematic highlights the terminals of the potentiostat connected to each driving or reference electrode, the build-up of electrochemical double-layer at each electrode surface and the redox reactions at each pole of the bipolar electrode immersed in the aqueous and organic electrolyte solutions, and the redox reactions taking place at the surface of each driving electrode to maintain electroneutrality in each phase at all times.[11]

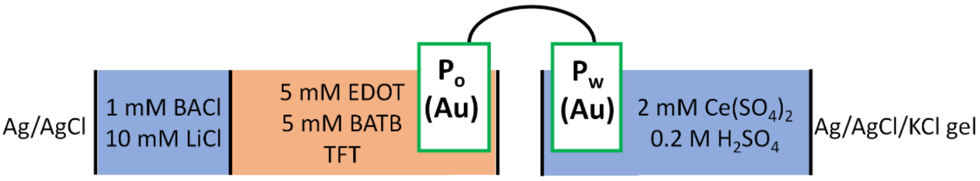


**Scheme S1. Schematic of the four-electrode CBPEC with aqueous|organic electrolyte solutions for the electropolymerisation of organic EDOT by aqueous $Ce^{4+}$.** The bipolar electrode consisted of two Au disc electrodes (CH Instruments, USA) connected by an electric

wire, one in each compartment acting as the aqueous pole ($P_w$) or organic pole ($P_o$), respectively. The measured potential was calibrated onto the Galvani potential scale using the procedure described above and elsewhere.[14] Before use, two individual pole Au disc electrodes were polished and cleaned electrochemically in 0.2 M $H_2SO_4$. The organic pole was also rinsed with de-ionised water, acetone and TFT solvent prior to CBPEC electrochemistry. Experiments were performed in ambient conditions.

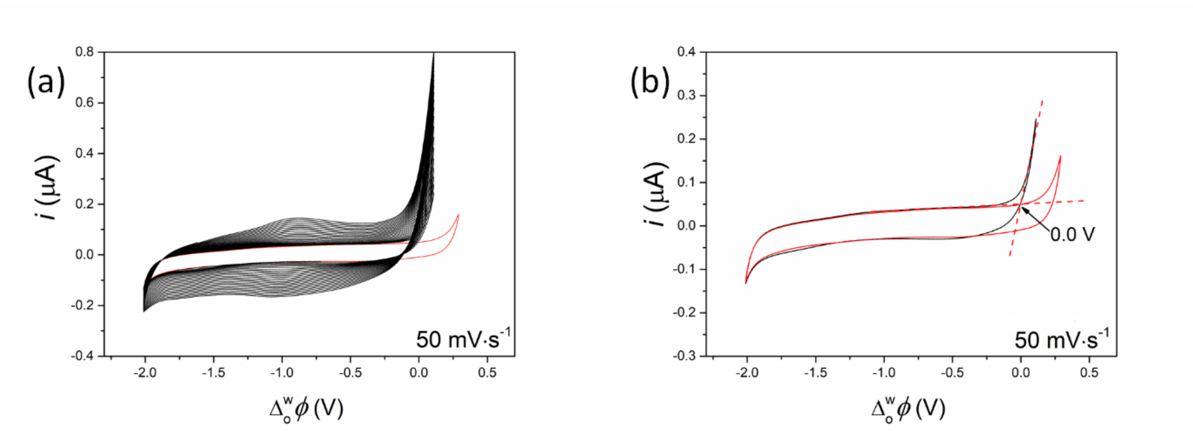


**Figure S8. Electropolymerisation of organic EDOT by aqueous $Ce^{4+}$ using a closed bipolar electrochemical cell (CBPEC) in a 4-electrode configuration.** (a) Repetitive cyclic voltammetry (CV) cycling using the CBPEC configuration outlined in Scheme S1. Experiments were performed in ambient conditions at a scan rate of 50 $mV{\cdot}s^{-1}$. Since the ET reaction between $Ce^{4+}$ and EDOT could take place at open circuit potential (OCP), the interfacial Galvani potential difference ($\Delta_o^w\phi$) was maintained at –1.5 V during the addition of EDOT to the organic phase to ensure no ET reaction took place prior to CBPEC cyclic voltammetry. Without EDOT present (red CV), the increase in current at ca. +0.2 V corresponds to electron transfer along the BPE from $P_o$ to $P_w$ as $P_o$ (a Au disc electrode) is oxidised and $Ce^{4+}$ is reduced at $P_w$. (b) For clarity, to show that $\Delta_o^w\phi_{IET}^{\ominus}$ for biphasic PEDOT electrosynthesis was ca. 0 V, the first scan in the presence of EDOT (black CV) is compared to a CV scan in the absence of EDOT (red trace) using the CBPEC configuration outlined in Scheme S1.

## Section S4. Electrochemically initiating, controlling, and monitoring PEDOT thin film interfacial electrosynthesis

### 4.1 Trends in the kinetics of interfacial electrosynthesis with double potential step chronoamperometry (DPSCA) cycle number

The charge recorded from both the positive IET and negative interfacial oligomer adsorption current transients during the first 18 DPSCA cycles display identical trends, as shown in Figure 2e (also see Figure S9 for details on how the charge was calculated). The charge increases very slowly for the first 5 DPSCA cycles, representing an initial induction period during which IET between $Ce^{4+}$ and EDOT is taking place at a bare ITIES (Figure 1a(i)), prior to the EDOT oligomers reaching the critical size necessary to adsorb (Figure 1a(ii)). Once the EDOT oligomers adsorb at DPSCA cycle 5 or 6, IET proceeds autocatalytically (Figure 1a(iii)) leading to PEDOT "islands" showing rapid 2D growth, parallel to the L|L interface during DPSCA cycles 6 to 9. By DPSCA cycle 9 or 10, a highly compact 2D PEDOT thin film has coalesced at the ITIES (Figure 1a(iv)) that acts a physical barrier between the $Ce^{4+}$ and EDOT species. Nevertheless, IET continues through the conductive PEDOT thin film for all DPSCA cycles > 9 as the thickness of the PEDOT thin film increases (Figure 1a(v)).

A key point to clarify is that from DPSCA cycle 9 or 10 onwards, the current initially stabilises and then only increases very moderately with further DPSCA cycling. This is due the highly compact 2D PEDOT thin film forming a more stable system, controlled by either mass transport or electron transfer, than the scenario with PEDOT "islands" at the interface during earlier DPSCA cycles. In other words, the presence of the highly compact 2D PEDOT thin film enables the entirety of the L|L interface to reach a similar potential distribution and, therefore, a stable current results. The autocatalytic effect continues through the conductive PEDOT thin film for all DPSCA cycles > 9, but the 4-electrode electrochemical cell cannot measure this

effect as the thin film grows far in excess of 1 nm in thickness, *i.e.*, far in excess of the width of the ITIES.

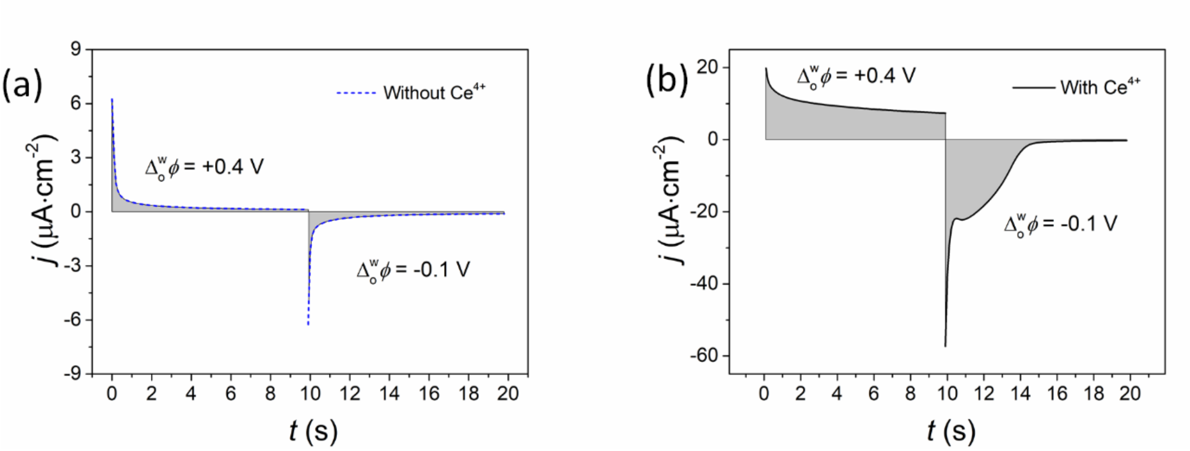


**Figure S9. Calculation of the charge generated during each DPSCA cycle of PEDOT interfacial electrosynthesis** using the 4-electrode electrochemical cell configuration outlined in Figure 2a (a) with (black lines) and (b) without (dashed blue lines) the aqueous $Ce^{4+}$ oxidation present. In each case, the shaded area under the current-time transient was integrated to calculate the background charge using Origin Pro 8.5.

The shapes of the interfacial adsorption current transients for the initial DPSCA cycles in Figure 2b are highly reminiscent of those recorded at solid electrode-electrolyte interfaces during nucleation and growth processes, for example, the underpotential deposition of Cu on Au(111) electrodes,[15] and conformational relaxation studies of doping in CP films.[16] As the interfacial oligomer adsorption current transients correspond to an interfacial ion-pairing reaction as EDOT oligomers assemble at the bare ITIES, their analysis will open opportunities to probe the kinetics of PEDOT nucleation and growth at the beginning of the interfacial electrosynthesis process. Furthermore, as noted *vide supra*, the nucleation and growth process at the ITIES may be probed independently of the IET step at different potentials. Such analysis is not possible for electropolymerisation at solid electrode electrolyte-interfaces, where the electron transfer and deposition steps take place simultaneously during anodic monomer

oxidation. An in-depth kinetic analysis of the DPSCA cycles is beyond the scope of this article, and a follow-on study is currently underway to develop a model to describe the mechanism of nucleation and growth of a PEDOT thin film at the ITIES as a function of the oxidant and monomer concentrations, applied $\Delta_o^w\phi$, and the nature of the aqueous electrolyte anion.

## 4.2 Interfacial ion-pairing and interchange between adsorbed cationic EDOT oligomers and aqueous $SO_4^{2-}$ anions

Evidence of interfacial ion-pairing and interchange between adsorbed cationic EDOT oligomers and aqueous $SO_4^{2-}$ anions during interfacial electrosynthesis is provided by the comparison of differential capacitance measurements of the blank aqueous|TFT interface and DPSCA cycles 2 to 6 in the presence of aqueous $Ce^{4+}$ and organic EDOT (Figure 2c). By DPSCA cycle 2, the potential of zero charge (PZC) shifts negatively from 0 to –0.055 V and the differential capacitance curve flattens. The PZC is sensitive to the interfacial adsorption of species either from the aqueous or organic phase; a negative shifting implies the adsorption of positive species.[17–19] Thus, the negative shift in PZC by DPSCA cycle 2 in Figure 2c is attributed to the interfacial adsorption of cationic EDOT oligomers. As noted in the main text, EDOT oligomers have a net cationic charge due to the weak coordination of the bulky $TB^-$ anions. The flattening of the curves indicates a decrease in permittivity at the aqueous|TFT interface due to the presence of these adsorbed oligomers.[20] With additional DPSCA cycles, a sharp peak appears centred at –0.01 V and is attributed to interfacial ion-pairing between the EDOT oligomers and aqueous $SO_4^{2-}$ anions, and simultaneous interchange of $TB^-$ and $SO_4^{2-}$ anions as the “dopant” anion. This peak’s magnitude increases with cycling as the EDOT oligomer concentration increases in the diffusion zone on the organic side of the ITIES. Also, the differential capacitance increases across the full width of the polarisable potential window with cycling due to accumulation of more charge as the PEDOT thin film grows.

## 4.3 *In situ* parallel beam UV/vis absorbance measurements

*In situ* parallel beam UV/vis absorbance measurements (experimental setup shown in Figure S10) were used to qualitatively monitor the steady reduction of $Ce^{4+}$ to $Ce^{3+}$ just above the L|L interface during interfacial electrosynthesis by following the decrease in absorbance at 385 nm with time (Figure S11a). Additional UV/vis absorbance measurements with the parallel beam shining through the PEDOT thin film confirmed that PEDOT is in an oxidised state at the ITIES (Figure S11b). The UV/vis spectrum was that of a *p*-doped PEDOT thin film,[21] with a polaronic band observed at ~700 nm.

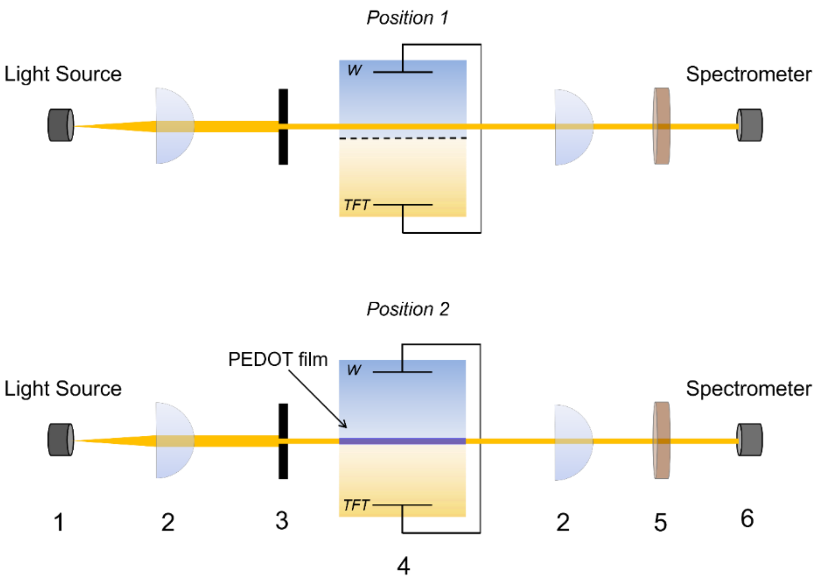


**Figure S10. Illustration of the optical setup for *in situ* parallel beam UV/vis absorbance measurements using a four-electrode electrochemical cell.** 1) DH-2000-BAL deuterium–halogen light source (Ocean Optics), 2) ultraviolet fused silica (UVFS) coated plano convex lenses (Thorlabs), 3) iris diaphragm (Thorlabs), 4) electrochemical cell, 5) neutral density (ND) filter (Thorlabs) and 6) Maya 2000 Pro Spectrometer (Ocean Optics). The electrochemical cell was moved to "*position 1*" for spectroscopic analysis of the aqueous phase, and "*position 2*" for analysis of the PEDOT film at the aqueous|TFT interface. The interfacial Galvani potential difference ($\Delta_o^w \phi$) was controlled using an Autolab PGSTAT204N potentiostat (Metrohm, Switzerland).

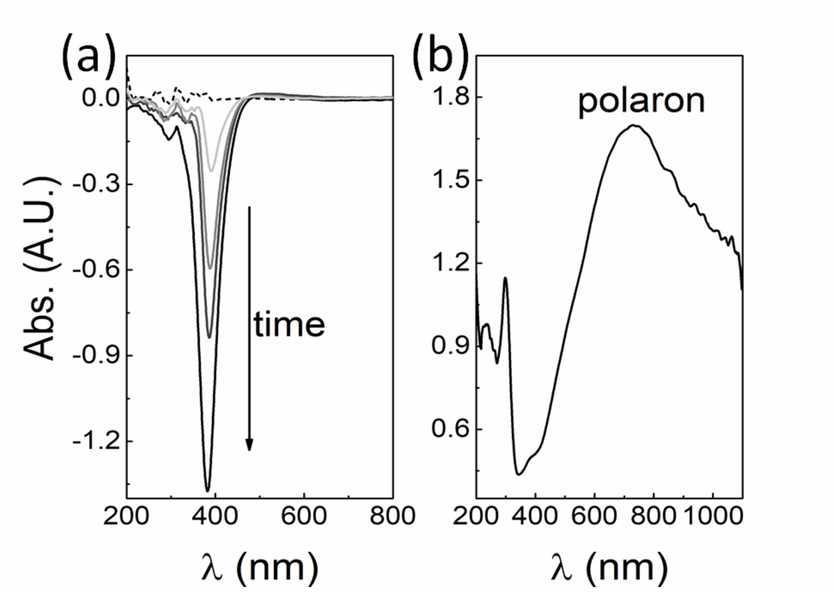


**Figure S11.** ***In situ*** **parallel-beam UV/vis absorbance measurements** of (a) the depletion of $Ce^{4+}$ on the aqueous side of the L|L interface with time (after 0, 15, 25, 35, 55 min.) during continuous DPSCA cycling and (b) the PEDOT thin film formed at the ITIES after 300 DPSCA cycles. All electrochemical experiments were performed using the cell configuration outlined in Figure 2a under aerobic conditions.

### 4.4 Using the Tyndall effect to explore the partition of small EDOT oligomers

After extended interfacial electrosynthesis (300 DPSCA cycles), a red laser beam was directed upwards through the four-electrode electrochemical cell, from bottom to top. A clear Tyndall effect was observed in both the organic and aqueous phase, attributed to EDOT oligomers' interaction in each phase with the laser light (Figure S12(a)). Thus, some EDOT oligomers partition or undergo ion transfer to the aqueous phase during interfacial electrosynthesis. To confirm this, the aqueous phase was recovered and cyclic voltammetry (CV) performed at a fresh ITIES formed between the recovered aqueous phase and a new organic phase (TFT containing 5 mM BATB electrolyte). An ion transfer signal at ca. 0.15 V was observed and attributed to ion transfer of small EDOT oligomers (Figure S12(b)). These oligomers are not expected to be involved in PEDOT thin film formation as the polymerisation reaction is

terminated very quickly in the aqueous phase due to a lack of monomer and high oxidant concentration, as seen previously for a polyaniline biphasic system,[22] precluding oligomer growth in the aqueous phase to reach the required critical size for adsorption. This assertion that the aqueous EDOT oligomers remain suspended in solution is supported by SEM images of the aqueous side of the PEDOT thin film (Figure S14(a) *vide infra*) that shows a flat and featureless surface.

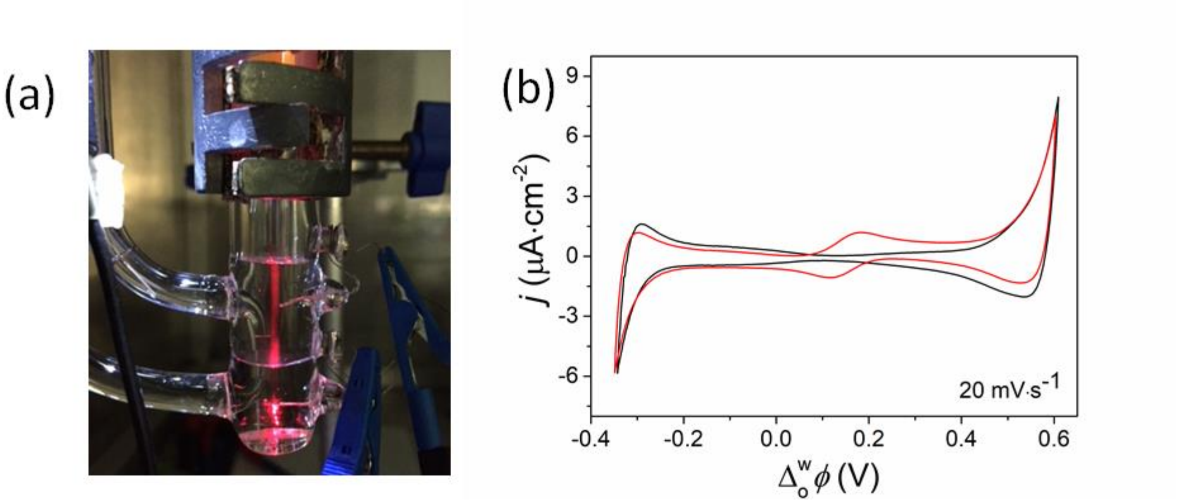


**Figure S12. Partition of small EDOT oligomers from the organic phase to the aqueous phase during interfacial electrosynthesis.** The latter is evidenced by (a) the Tyndall effect being observed in both phases in the four-electrode electrochemical cell after interfacial electrosynthesis and (b) observation of a clear ion transfer signal by cyclic voltammetry (CV) at an ITIES formed between an aqueous phase recovered after interfacial electrosynthesis and a freshly prepared organic phase (TFT containing 5 mM BATB electrolyte). The ion transfer signal at 0.15 V is attributed to small EDOT oligomers present in the aqueous phase and the scan rate used was 20 mV·s$^{-1}$.

## 4.5 Potentiostatic polarisation of the L|L interface

A current-time transient recorded during potentiostatic interfacial electrosynthesis using the four-electrode electrochemical cell described in Figure 2(a) is shown in Figure S13. $\Delta_o^w\phi$ was set to +0.4 V, an optimal potential to induce IET between the organic EDOT monomers and aqueous $Ce^{4+}$, and applied for 1,000 s. The current density initially decayed and reached a current minimum after ∼50 s. Following this decay, the current density increased to a plateau and then decreased slightly. The increase in current density corresponds to the growth of the PEDOT thin film at the L|L interface. However, the magnitude of the current density remains very low throughout the electrosynthesis (0.2 – 1.5 $\mu A \cdot cm^{-2}$), compared to a current density of ~10 $\mu A \cdot cm^{-2}$ for the +0.4 V step during DPSCA (Figure 2d). This indicates that the quantity of EDOT oligomers deposited at the L|L interface throughout the potentiostatic interfacial electrosynthesis is minimal.

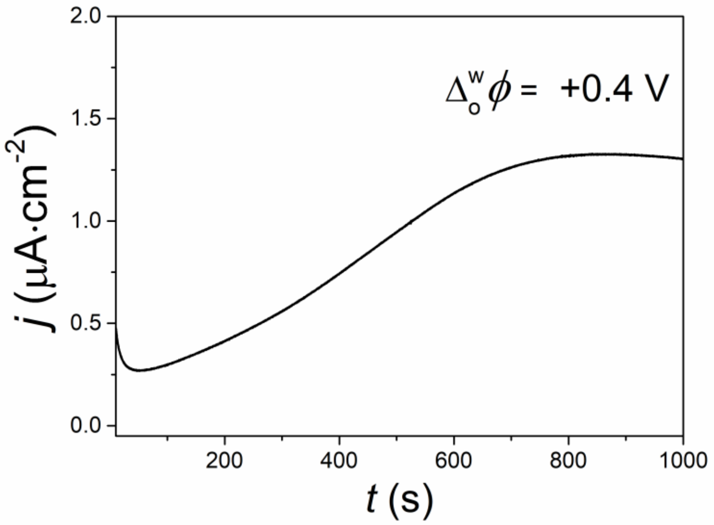


**Figure S13. Current-time transient recorded during potentiostatic interfacial electrosynthesis using the cell configuration outlined in Figure 2a under aerobic conditions.** $\Delta_o^w\phi$ was set to +0.4 V, optimal to induce IET between the organic EDOT monomers and aqueous $Ce^{4+}$, and applied for 1,000 s.

## Section S5. Microscopic analysis of the PEDOT thin film

Scanning electron microscopy (SEM) and transmission electron microscopy (TEM) samples were prepared by drop-casting PEDOT thin films onto Cu mesh grids, as shown by SEM images in Figures S14-16. SEM measurements were performed with a Thermo Scientific™ Helios™ G4 CX DualBeam™ microscope at 5 kV using a Through-the-Lens Detector (TLD) in immersion mode. TEM measurements were performed at 80 kV with a ThermoFisher USA (formerly FEI Co.) Titan Themis Z (60−300 kV) TEM equipped with a double Cs (spherical aberration) corrector, a high brightness electron gun (xFEG), an electron beam monochromator, and a Gatan Quantum 966 imaging filter. Atomic force microscopy (AFM) was undertaken in tapping mode using a Pt-coated Si tip (PPP-EFM, Nanosensors, Switzerland) with a stiffness constant of 2.8 $N \cdot m^{-1}$ and resonance frequency of 67 kHz on an MFP-3D infinity AFM system (Asylum Research, USA). Contact angle measurements were made on both sides of the PEDOT thin films using a Theta Attension Optical Tensiometer from Biolin Scientific (Sweden) controlled using OneAttension software version 2.0 (see Figure 3b). Water contact angle measurements were performed with Milli-Q deionized water (18.2 $M\Omega \cdot cm$) at RT (21°C). A droplet volume of 10 μL was used.

### 5.1 Scanning electron microscopy (SEM) analysis

SEM of a 2D PEDOT thin film prepared by DPSCA cycling using the cell configuration in Figure 2a revealed an asymmetric “Janus” morphology (Figure 3a and Figure S14). The flat side is the aqueous facing side and mirrors the defect-free nature of the L|L interface. The rough side is the organic facing side and resembles the morphology of PEDOT, and many other CPs, typically observed after electropolymerisation at conventional solid electrode-electrolyte interfaces.

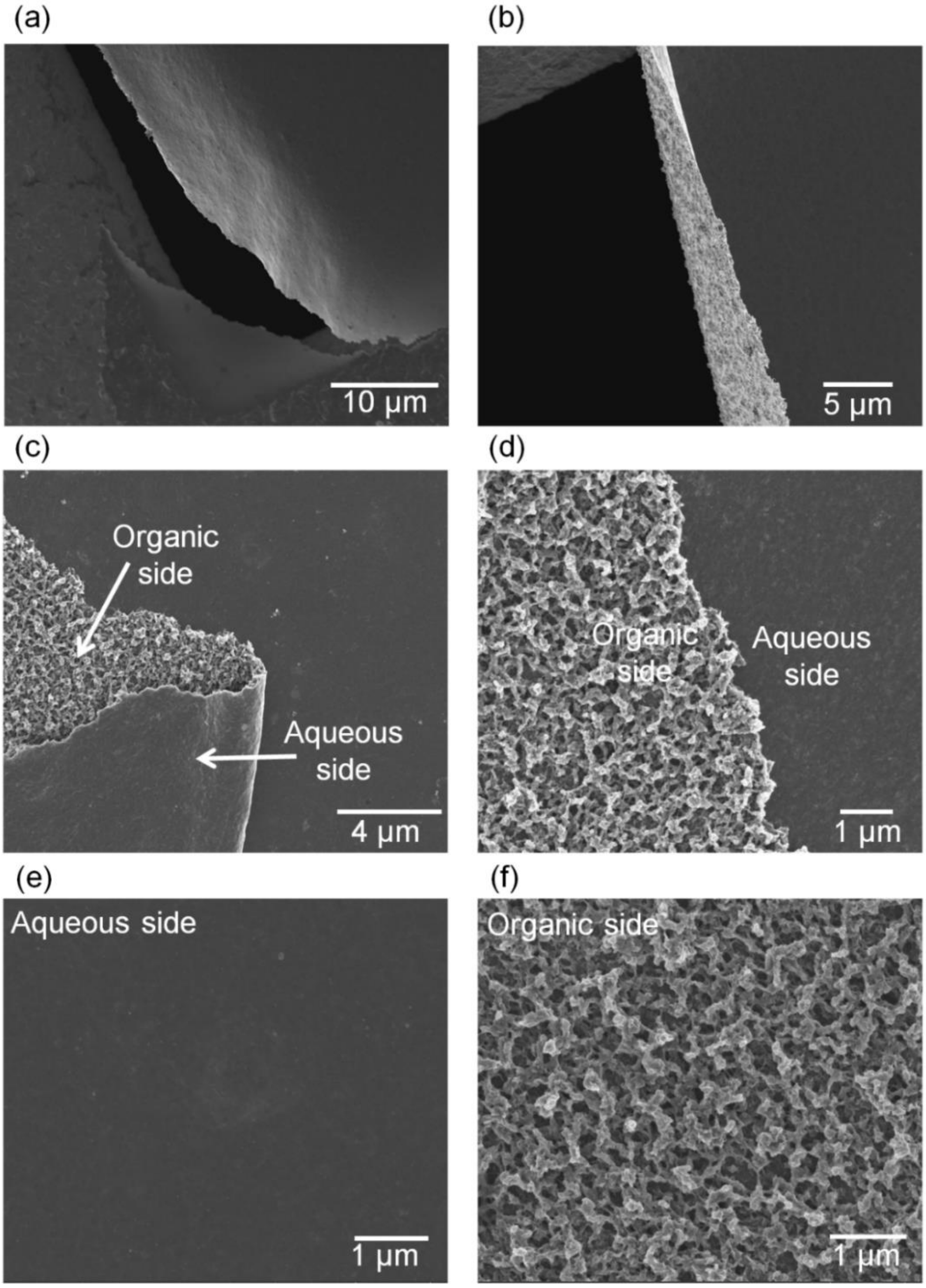


**Figure S14. Scanning electron microscopy (SEM) of 2D PEDOT thin films prepared by DPSCA using the cell configuration in Figure 2a.** The asymmetric "Janus" morphology is clear in SEM images (a) to (d). SEM images (e) and (f) clearly demonstrate that the aqueous-facing side is flat and featureless at the nanoscale, while the organic facing-side shows a rough, porous 3D structure, respectively. The PEDOT thin film shown in (a) and (b) was prepared using 150 DPSCA cycles, and the film shown in (c) – (f) was prepared using 300 DPSCA cycles.

This asymmetric morphology confirms that any small EDOT oligomers that undergo partition or ion transfer across the ITIES to the aqueous phase are not involved in thin-film formation. Instead, the L|L interface acts as an anchoring point and upon IET during DPSCA cycling, the PEDOT thin film nucleates and grows “down” into the organic phase. Furthermore, the PEDOT thin films adhere well to solid substrates, closely following the contours and taking the shape of the surface (see Figures S14 and S15).

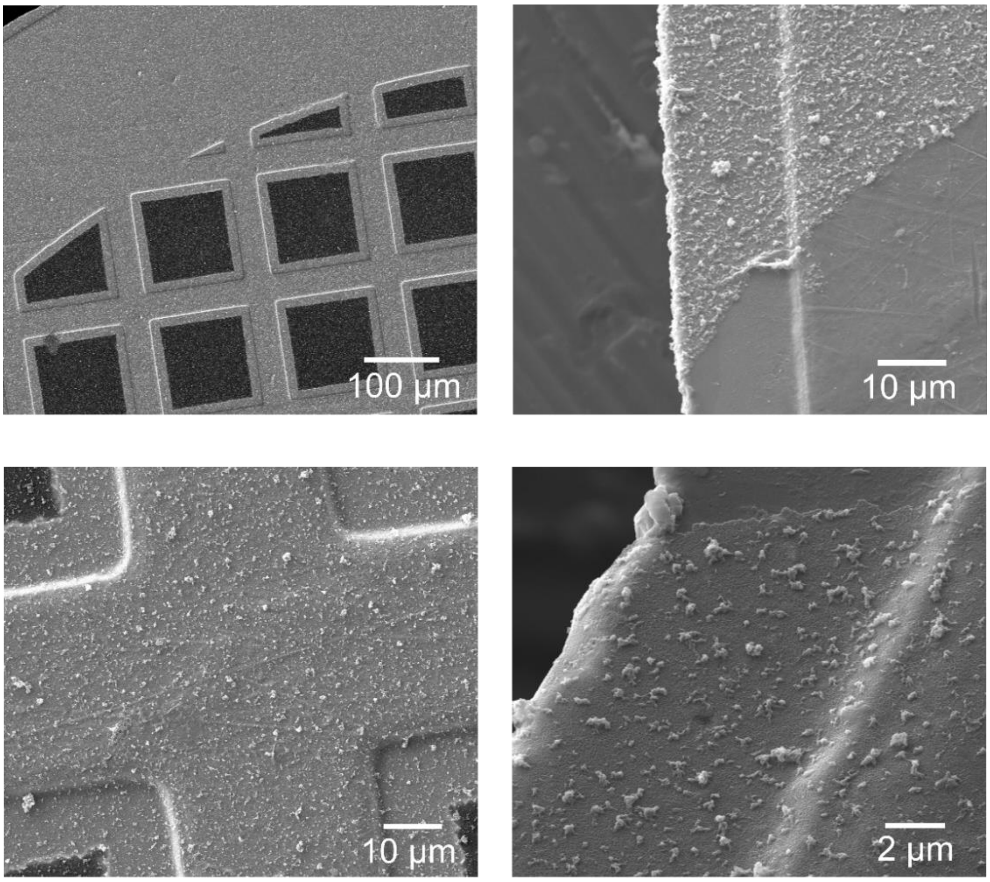


**Figure S15. Strong adhesion of the PEDOT thin film to a solid substrate, with the thin film closely following the contours and taking the shape of the surface.** SEM image of a PEDOT thin film with the flat and featureless aqueous side facing down after being drop cast onto a Cu mesh grid for TEM analysis.

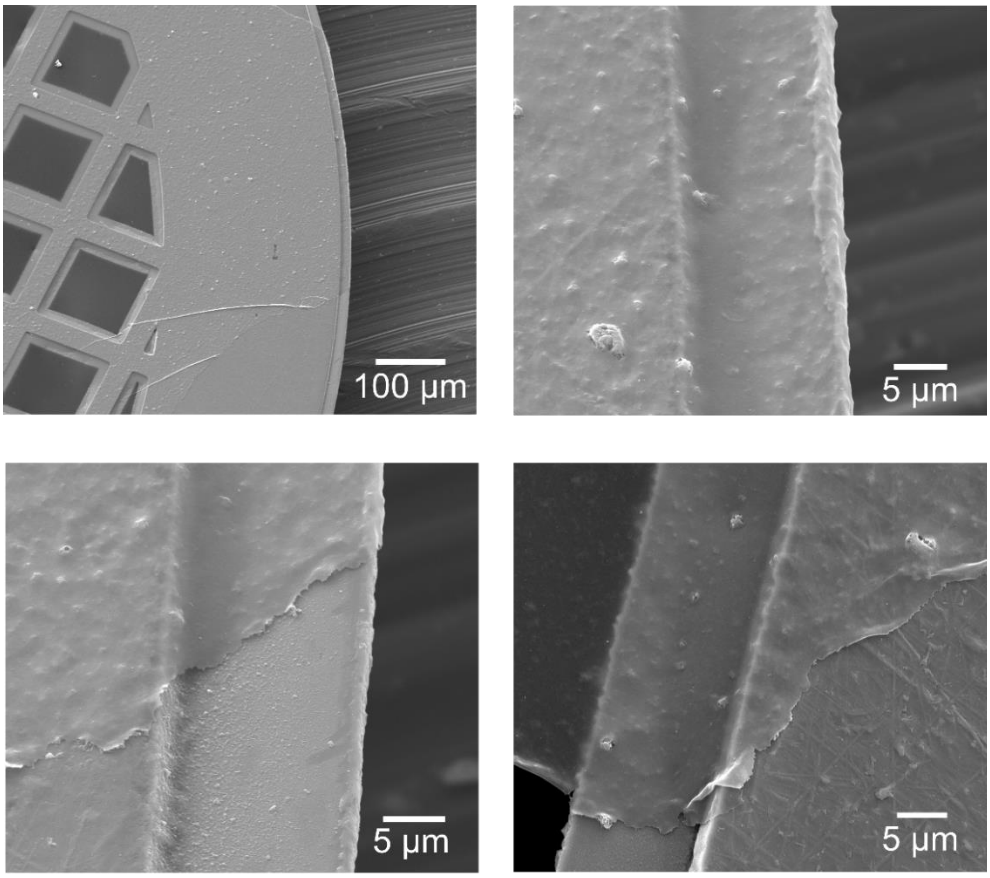


**Figure S16. Strong adhesion of the PEDOT thin film to a solid substrate, with the thin film closely following the contours and taking the shape of the surface.** SEM image of a PEDOT thin film with the rough organic side facing down after being drop cast onto a Cu mesh grid for TEM analysis.

### 5.2 Atomic force microscopy (AFM) analysis

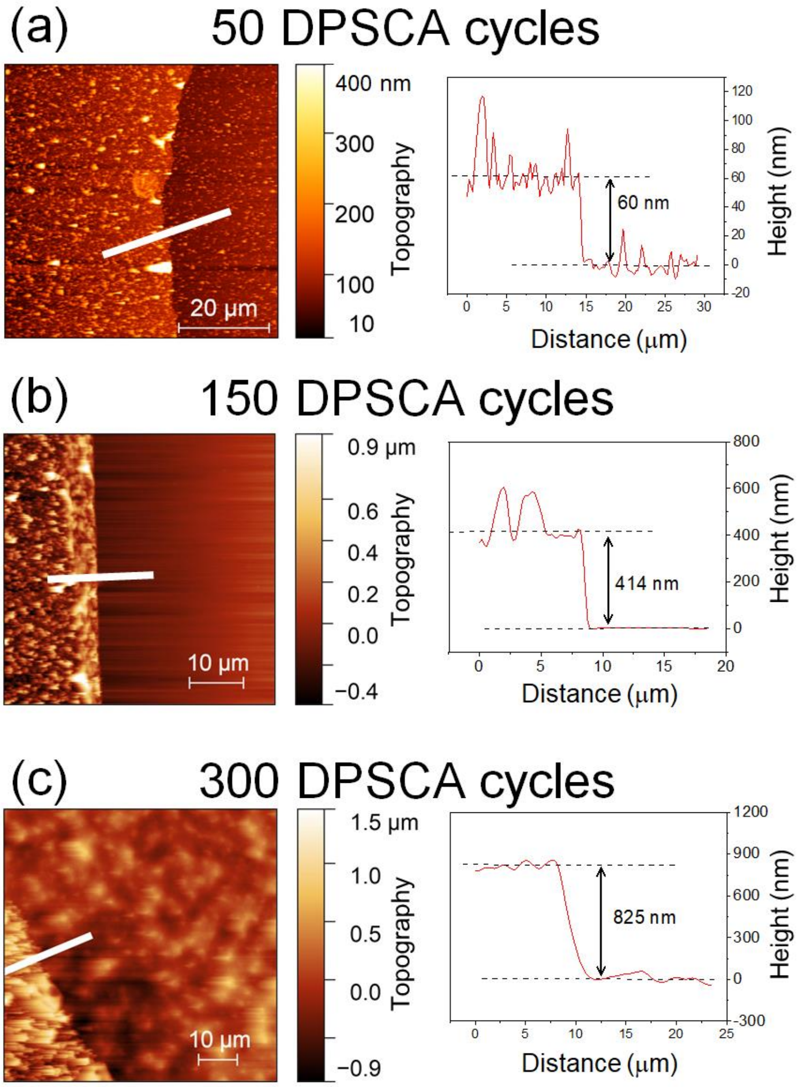


**Figure S17. Atomic force microscopy (AFM) determination of the thickness (*t*) of PEDOT thin films** on a flat gold substrate as a function of the number of DPSCA cycles during interfacial electrosynthesis. The corresponding SEM images of the organic facing sides of these PEDOT thin films are shown in Figure 3e.

## Section S6. Spectroscopic analysis of the PEDOT thin films

PEDOT thin films were recovered from the aqueous|TFT interface, washed, and then drop cast onto an ITO glass slide for UV/vis-NIR absorbance analysis, a stainless-steel specimen disc for Raman analysis and a polished silicon wafer substrate for X-ray-photoelectron spectroscopy (XPS) analysis. UV/vis-NIR absorbance measurements were made using a DH-2000-BAL deuterium–halogen light source and a Maya 2000 Pro Spectrometer. Raman spectroscopic measurements were performed using a LabRAM HR Evolution Raman Confocal Microscope (Horiba, France) with LabSpec 6 software. Measurements were performed with a 783 nm excitation line laser source. Calibration was performed with a Silicon standard (520.07 $cm^{-1}$). XPS measurements were performed using a Kratos ULTRA spectrometer (Kratos Analytical, UK) with monochromatic Al Kα radiation energy of 1486.58 eV. C 1s at 284.8 eV was used as the charge reference to determine the core level binding energies.

### 6.1 UV/vis-NIR absorbance

The UV/vis-NIR absorbance spectrum displays a broad absorption that extends into the infrared region due to bipolaron band formation (Figure S18).[23,24] The presence of the bipolaron band signifies that the PEDOT thin film is in an oxidised state[25] and *p*-doped during interfacial electrosynthesis, in agreement with the *in situ* UV/vis-NIR absorbance spectrum *vide supra* (see Figure S11b).

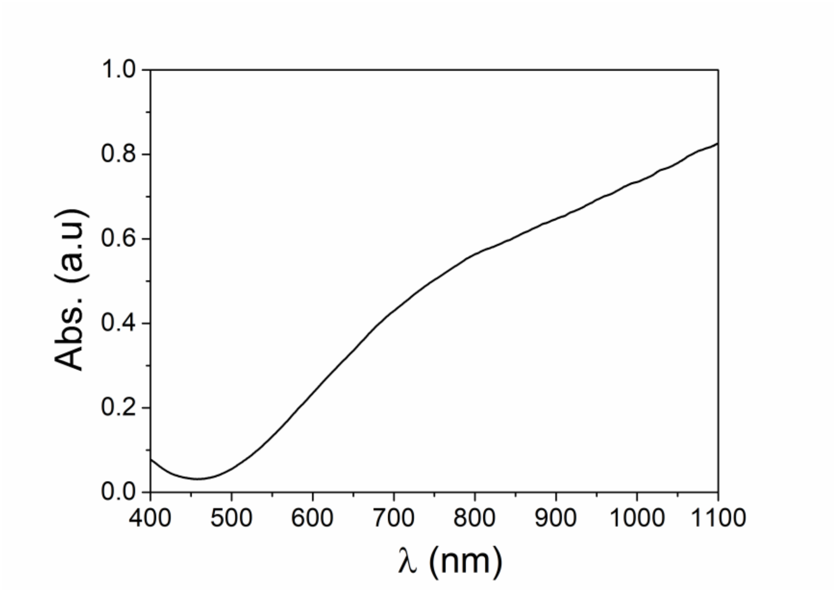


**Figure S18.** UV/Vis-NIR absorbance spectrum of a PEDOT thin film immobilised on an ITO glass slide.

## 6.2 Raman spectroscopy

Several *in situ* Raman spectroelectrochemical studies have shown that the benzenoid configuration is the more stable form when a PEDOT film is highly doped.[21,26,27] In this work, the Raman spectrum (obtained using excitation from a red laser, $\lambda = 785$ nm) shows a dominant peak at 1425 cm$^{-1}$ attributed to the characteristic band of symmetric $C_\alpha{=}C_\beta$ stretching (Figure S19). The relatively high wavenumber of 1425 cm$^{-1}$ for this $C_\alpha{=}C_\beta$ stretching implies that the PEDOT thin-film is highly *p*-doped with a benzenoid (coiled) configuration to the polymer chain.[28,29] Several peaks characteristic of PEDOT films were observed. For example, the shoulder peaks at 1365 cm$^{-1}$ and 1531 cm$^{-1}$ are attributed to asymmetric $C_\alpha{=}C_\beta$ stretching and $C_\beta$–$C_\beta$ stretching, respectively.[21,26] The peak at 1255 cm$^{-1}$ can be assigned to inter-ring $C_\alpha$–$C_{\alpha'}$ stretching.[21,27]

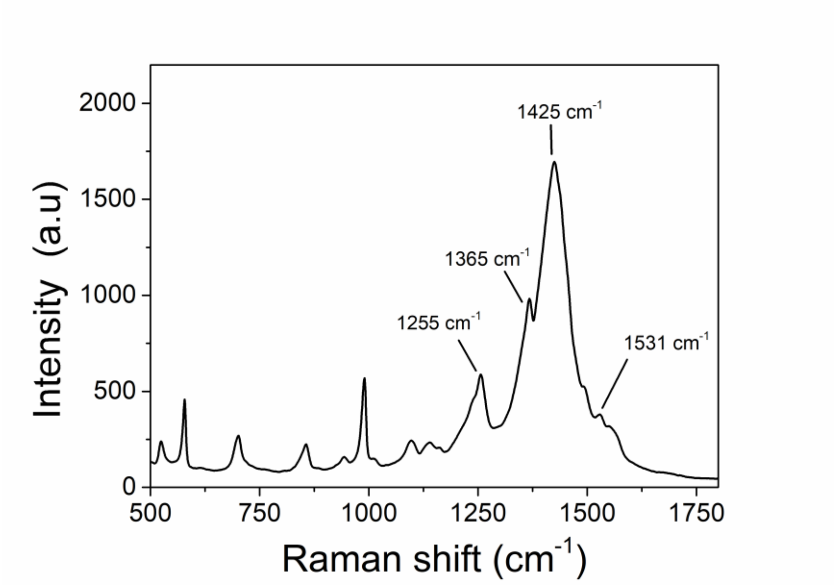


**Figure S19.** Raman spectrum of the PEDOT thin film immobilised on a stainless-steel specimen disc using an excitation laser wavelength of 785 nm.

### 6.3 X-ray photoelectron spectroscopy (XPS)

XPS was used to probe the chemical composition and doping level of the PEDOT thin film.[30] The XPS survey spectrum shows the presence of only S, C and O within approx. 10 nm sampling depth (Figure S20), indicating that the Ce oxidant is not incorporated into the thin film during interfacial electrosynthesis. Oxidant contamination is a common drawback associated with traditional chemical polymerisation methods, which must be addressed with post-processing purification.[31–33] Furthermore, the absence of Ce from the PEDOT thin films prepared herein is expected to enhance cell biocompatibility (as demonstrated using biocompatibility studies in the main text and Section S8). The absence of boron and fluorine implies that the organic electrolyte $TB^-$ anion is not involved in the *p*-doping of the thin film. Therefore, the aqueous $SO_4^{2-}$ anion is considered the primary dopant. Analysis of both sides of the thin film showed the same chemical composition (Figure S20).

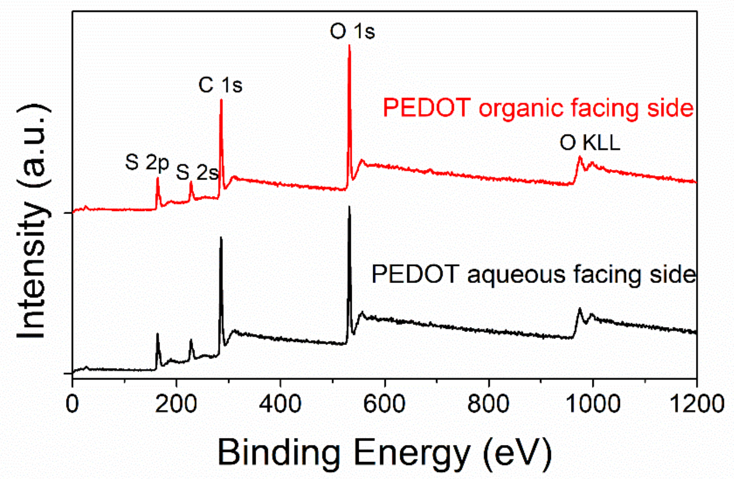


**Figure S20.** X-ray Photoelectron Spectroscopy (XPS) survey spectra of the two different sides of the PEDOT thin film (with the PEDOT thin-film immobilised on a silicon wafer substrate).

The doping level of the PEDOT film was estimated by analysis of the high resolution S 2p spectrum (Figure S21a).[34] The sulfur atom present in each EDOT unit can be distinguished from the sulfur atom in each $SO_4^{2-}$ anion by the differences in their binding energies.[35,36] The S 2p doublet from the $SO_4^{2-}$ anion appears at higher binding energies (168 – 170 eV) due to the presence of four electronegative oxygen atoms withdrawing electron density from the sulfur atom. In comparison, the sulfur present in the thiophene ring of EDOT occurs at approx. 164.0 eV. Therefore, comparing the peak areas attributed to each sulfur species allowed a direct estimation of the doping levels as ~39% within a 10 nm sampling depth of the PEDOT thin film, in line with previous reports of the upper limits possible of PEDOT doping between 35 – 40%.[37,38]

The deconvoluted C 1s spectra for the PEDOT thin film is shown in Figure S21b. The peak at 284.8 eV corresponds to both C–C and C=C bonding within the PEDOT structure, while the peak at 285.7 eV can be assigned to C–S bonds.[39] The peak at 286.4 eV is due to the conjugated C=C–O bond, and the peak centred at 287.4 eV corresponds to the ethylene ring's

C–O–C bond.[29] Evidence of high conjugation within the film can be seen from the intensity of the π→π* shake up transition ~291 eV.[35,40]

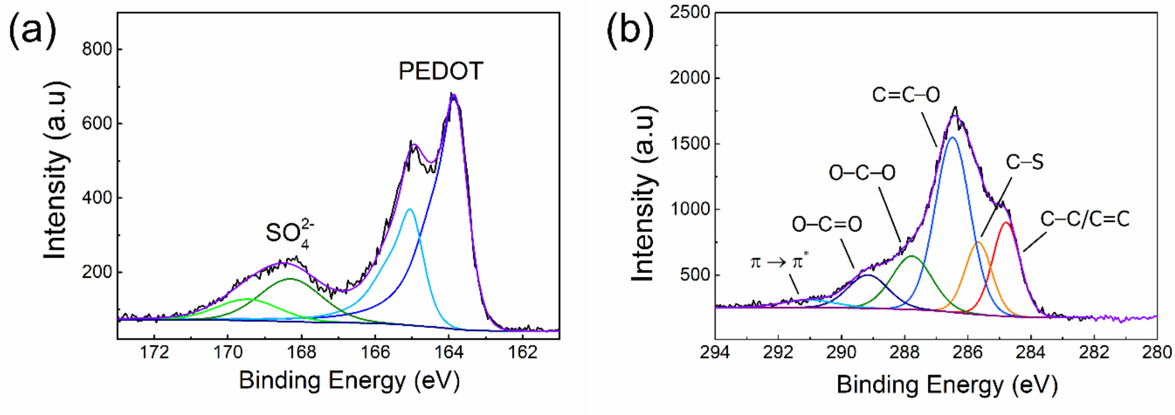


**Figure S21.** XPS of the PEDOT film PEDOT film immobilised on a silicon wafer substrate: (a) the high resolution S 2p spectrum and (b) the C 1s spectrum.

## Section S7. Conductivity and electrochemical properties of the PEDOT thin film

### 7.1 The impact of excess PSS on conductivity, long-term stability and specific capacity

The anionic PSS surfactant enhances the processability of hydrophobic PEDOT dispersions by increasing their solubility in aqueous solution.[41] PSS also becomes the primary dopant in the polymer, neutralising the positive charges of oxidised PEDOT during synthesis. Excess negative charge from the PSS can alter the conduction mechanism of the polymer film.[42] Indeed, an excess of PSS has detrimental effects on CP thin-film conductivity, long-term stability and specific capacity.[43–46] The field has attempted to overcome these negative contributions of PSS through post-film deposition treatments, such as chemical capping with the (3-glycidyloxypropyl)trimethoxysilane (GOPS) crosslinker or acid washing to reduce PSS content, with variable outcomes[47,48] and often compromised PEDOT conductivity.[49] In photovoltaic applications, PEDOT:PSS is widely known to cause device degradation and instability due to the acidity of the PSS chain. Sulfonic acid groups on the PSS chain interact with moisture from the atmosphere, resulting in an acidic solution that can breakdown PEDOT chains or degrade the indium tin oxide (ITO) transparent electrode.[50] Therefore, it is highly desirable to prepare pure PEDOT films, in the total absence of PSS surfactant.

### 7.2 *Ex situ* and *in situ* conductivity of the PEDOT thin film

A materials electrical conductivity is related to its performance in an electronic device.[51] *Ex situ* (in-plane or dry) conductivity ($\sigma_{Ex}$) of the PEDOT thin film prepared by interfacial electrosynthesis was determined with a four-strip conductivity electrode (ALS, Japan, see Figure S22) using the method described elsewhere[52–54] and herein. The four-strip method was favoured over the conventional four-point-probe setup due to the better contact achieved with

the former as the probe tips caused significant tearing of the PEDOT thin film sample. The conductivity electrode was cleaned using HPLC grade acetone and isopropanol prior to use. Once clean, the PEDOT thin film was drop cast across the four-strip conductivity electrode and oven dried at 50$^{o}$C for 2 hr. Very thin PEDOT thin films (approx. 50 nm) were used for these measurements, with flat morphologies on both sides of the film as shown in Figure 3e(i).

*Ex situ* conductivity measurements were performed with an Autolab PGSTAT204 potentiostat from Metrohm (Netherlands), controlled using NOVA software version 2.1.2, at RT (21$^{o}$C) in ambient conditions. A linear galvanostatic sweep up to 1 mA was applied between the two outer electrodes, while the potential between the two inner electrodes was monitored. *Ex-situ* conductivity ($\sigma_{Ex}$) was calculated using Equation S2, where *w* is the sample width, $t$ is the thickness of the sample (determined by AFM), and *l* is the distance between the distance between the two inner electrodes. The conductivity measurement was repeated for three different PEDOT thin films with the average value presented.

$$\sigma_{Ex} = \frac{I \cdot l}{V \cdot w \cdot t} \qquad \text{(S2)}$$

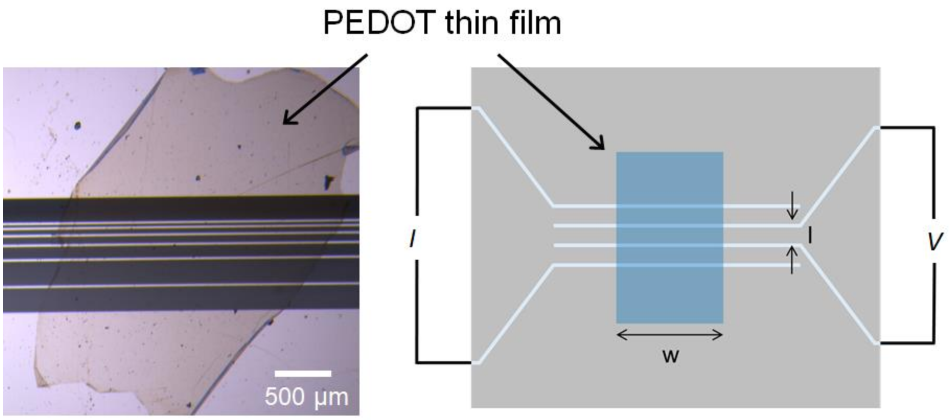


**Figure S22. Optical image and schematic illustration of the four-strip microelectrode array** from ALS, Japan used to obtain *ex situ* (in plane) conductivity measurements of PEDOT films. The image shows a PEDOT thin film prepared by interfacial electrosynthesis fully contacting all four strips.

A value of 554 (± 77) S·cm$^{-1}$ was determined, comparable to the highest conductivity value reported for a pristine PEDOT: $ClO_4^-$ film (400–650 S·cm$^{-1}$) made by conventional electropolymerisation at a solid electrode surface in acetonitrile.[55,56] Note that, the electrical conductivity could be improved or decreased depending on the dopant anion selected; for example, conventional electropolymerised PEDOT films doped with $SO_4^{2-}$ leads to a decrease in conductivity to 40 S·cm$^{-1}$.[33] The influence of the aqueous electrolyte on PEDOT interfacial electrosynthesis will be published in a forthcoming work.

PEDOT:PSS is the most widely used active layer material in organic electrochemical transistor (OECT) devices.[24,57–59] The operation of such devices is based on changing the polymer films' oxidation state or doping level and hence its conductivity.[60,61] OECT devices typically operate in depletion mode. In this scenario, the PEDOT:PSS film is initially in the conductive *p*-doped (or ON) state with current flow between the "source" and "drain" of the OECT device. The PEDOT:PSS film becomes insulating in the dedoped (or OFF) state when reduced to the neutral form, which stops the current flow between the source and 'drain'. Therefore, *in situ* conductivity measurements identify the precise potential range where the switching between the ON and OFF states takes place. Phosphate buffer saline (PBS) solution is widely used in OECT devices and was therefore chosen as the electrolyte for the *in situ* conductivity and electrochemical characterisation studies *vide infra*.

*In situ* conductivity ($\sigma_{IN}$) measurements were performed in PBS solution using an interdigitated microelectrode array (NanoSPR, USA) operated in a transistor-like configuration as illustrated in Figure S23, with a Pt wire counter electrode and Ag/AgCl (KCl gel) reference electrode. The microelectrode array was operated with a WaveDriver 20 bipotentiostat from Pine research (USA) controlled by AfterMath software version 1.6. PEDOT thin films prepared by interfacial electrosynthesis were drop cast onto the interdigitated microelectrode array, and oven dried at 60°C for 2 h. For the PEDOT:PSS films, a quantity of the PEDOT:PSS ink was

drop cast onto the electrode surface and dried for 2 h in the oven at 60°C. The thickness of the PEDOT films were determined by AFM prior to *in situ* conductivity measurement. *In situ* conductivity measurements were performed in a nitrogen environment.

The array has two microelectrodes, called the "source" and the "drain" which were both in contact with the PEDOT film. An "off-set" potential ($V_D$) of 10 mV was maintained between the two microelectrodes during the measurement. Simultaneously, the potential of the microelectrodes ($V_G$) *vs.* the Ag/AgCl (KCl gel) reference electrode was varied at a scan rate of 20 mV·s$^{-1}$, allowing the PEDOT film to transition between oxidation states. When the PEDOT film is conductive, significant drain current ($i_D$) flows between the "source" and the "drain". When the PEDOT film is insulating, the drain current is negligible. $i_D$ is directly proportional to the conductivity of the PEDOT thin film; thus, a plot of $i_D$ *vs.* $V_G$ provides relative conductivity *vs.* potential.[62] The current that flows between the PEDOT film and the counter electrode is the faradaic current ($i_F$). Equations (S3) and (S4) were used to calculate the values of $i_D$ and $i_F$.[63] Equation (S5) was used to convert $i_D$ into conductivty ($\sigma_{IN}$), where *d* is the distance between the two microelectrodes, *l* is the length of the polymer film on the microelectrode array (shown in Figure S23b), *n* is the number of spaces between electrodes and *t* is the thickness of the polymer film.[64] $\sigma_{IN}$ values determined using this method should not be considered absolute values as measurements are performed using a two probe method.[65]

$$i_D = (i_2 - i_1)/2 \qquad \text{(S3)}$$

$$i_F = (i_2 + i_1)/2 \qquad \text{(S4)}$$

$$\sigma_{IN} = \frac{i_D}{V_D}\frac{d}{n \cdot t \cdot l} \qquad \text{(S5)}$$

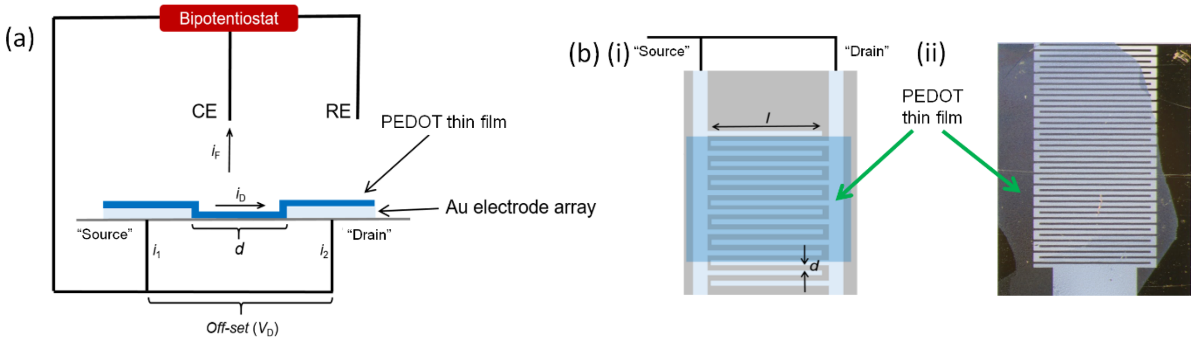


**Figure S23. Schematics of the experimental setup to carry out *in situ* conductivity measurements**. (a) Side view illustration and (b) top view illustration and image of the configuration of the Au interdigitated microelectrode array used to determine the *in situ* conductivity of PEDOT thin films, where *d* is the distance between the "source" and "drain" microelectrodes and *l* is the length of the polymer film.

A comparison of *in situ* conductivity measurements of a PEDOT thin film prepared by interfacial electrosynthesis (and doped with $SO_4^{2-}$) and a commercial PEDOT:PSS film dropcast and annealed directly onto the interdigitated microelectrode array is shown in Figure S24. *In situ* conductance was recorded from a positive potential, where the PEDOT is fully oxidised and conducting or ON, to a negative potential. Both PEDOT films displayed the typical sigmoidal response expected for CP films.[60] The PEDOT thin film had a maximum conductivity of ~5.35 S·cm$^{-1}$ in the plateau region from +0.8 to 0.0 V (*vs.* Ag/AgCl). The PEDOT thin film conductivity began to decrease from 0.0 V and was fully dedoped and insulating at –0.85 V. The PEDOT:PSS film had a maximum conductivity of ~1.2 S·cm$^{-1}$ in the plateau region from +0.8 to +0.3 V. The conductivity decreased towards negative potential, becoming fully dedoped at –0.45 V.[55] Note that, *in situ* conductivity measurements are made using a two-probe technique, and therefore the conductivity values obtained using this method should be considered relative to *ex situ* conductivity measurements which are more accurate.[63] The conductance window in the PEDOT thin film is +0.4 V greater than that of the

PEDOT:PSS film in PBS buffer solution. The incorporation of PSS into PEDOT films has previously been shown to alter their doping/dedoping potential.[24,66] The extended potential window seen with the PEDOT thin film prepared by interfacial electrosynthesis could have potential advantages in OECT devices such as working at lower potentials to avoid oxidative reactions or biological stress (if the active layer is to be functionalized with cells). Future work will focus on the influence of the dopant anion on the conductivity and active potential window of the polymer film measured by the *in situ* conductivity method.

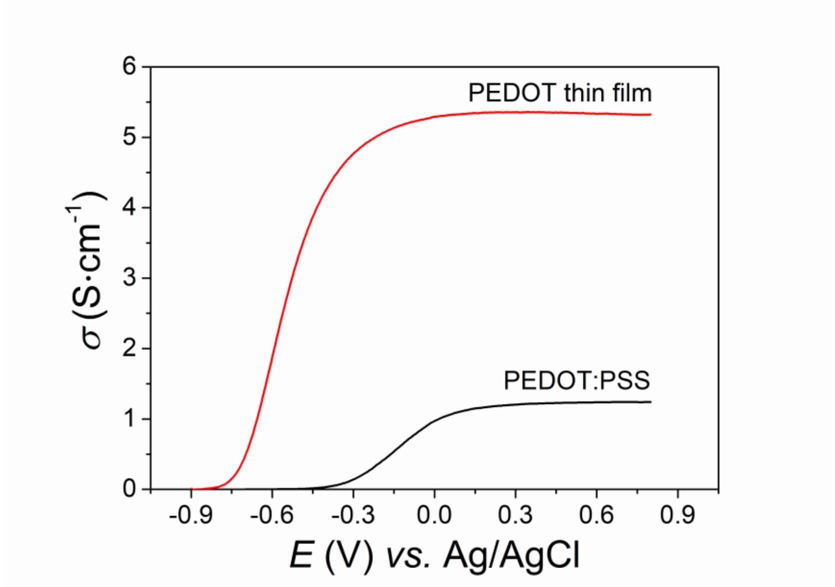


**Figure S24. *In situ* conductivity measurements of the PEDOT thin film prepared by interfacial electrosynthesis and a commercial 'high conductivity grade' PEDOT:PSS film in PBS solution.** The scan rate used was 20 mV·s$^{-1}$ and the offset potential ($V_D$) was 10 mV. The electrochemical cell bubbled with nitrogen for 30 mins before measurements.

## 7.3 Electrochemical characterisation of the PEDOT thin film in a 3-electrode configuration immobilised on a solid electrode surface

Electrochemical characterisation of PEDOT films was performed in PBS solution by cyclic voltammetry (CV) using a three-electrode configuration. The working electrode was either a

Au disc electrode or an indium tin oxide (ITO) glass slide (60 Ω). The working electrode was coated by the PEDOT film sample as described for the *in situ* conductivity experiments for PEDOT thin films prepared by interfacial electrosynthesis and the PEDOT:PSS films. Immobilised PEDOT films were rinsed with DI water and PBS solution before use. The counter and reference electrodes were a Pt wire and an Ag/AgCl (KCl gel) electrode, respectively. The Au electrode was used to investigate the electrochemical doping/undoping of the PEDOT films due to the Au electrodes wide potential window under anaerobic conditions (Figure S25). ITO electrodes were used to investigate the PEDOT films scan rate dependence under aerobic conditions (Figure S26).

CVs of the PEDOT thin film and PEDOT:PSS film in PBS buffer solution using a range of high scan rates between 0.2 and 5 $V{\cdot}s^{-1}$ are shown in Figure S25. Measurements were made in the potential range between –0.2 to +0.6 V (vs. Ag/AgCl). The CV's show the classical electrochemical response for conducting polymers films.[24,65] During the forward scan, the onset of oxidation takes place at –0.6 V (vs. Ag/AgCl) in the PEDOT:PSS film, and –0.85 V in the PEDOT thin film. Polymeric chains within the PEDOT films become positively charged upon oxidation, which are neutralized by counter anions ($Cl^-$, $H_2PO_4^-$ or $HPO_4^{2-}$) present in the PBS buffer solution. This charge compensation process is known as "p-doping" and leads to the observed increase in capacitance. A decrease in capacitance is observed during the backwards scan at –0.1 V in the PEDOT:PSS film and –0.7 V in the PEDOT thin film. This response corresponds to "dedoping" of the polymer film.

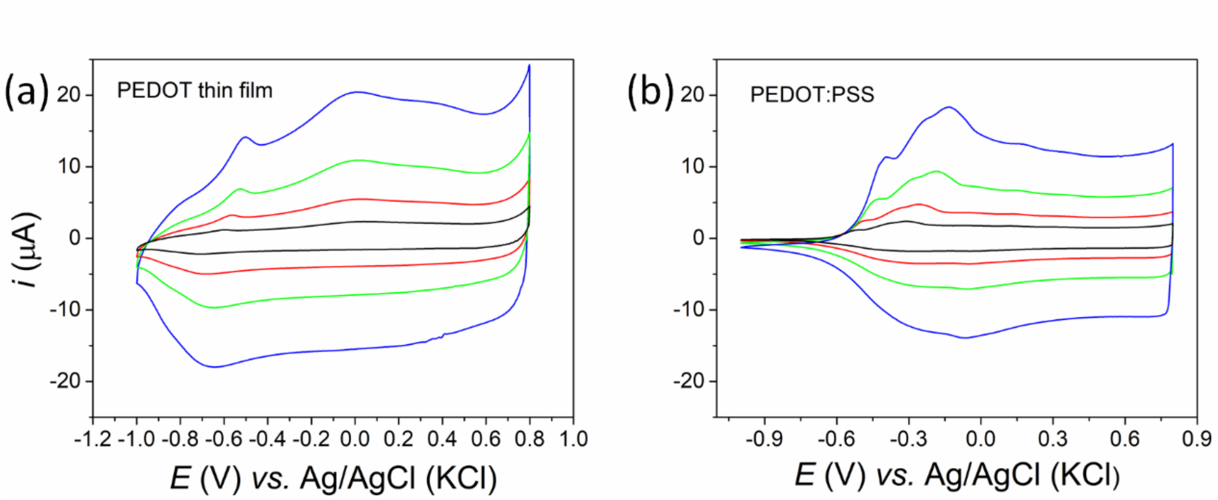


**Figure S25.** Cyclic voltammetry (CV) of (a) a PEDOT thin film prepared by interfacial electrosynthesis and (b) PEDOT:PSS film immobilised on Au electrodes in PBS solution at scan rates of 20 (black), 50 (red), 100 (green) and 200 (blue) mV·s$^{-1}$. CVs were obtained under anaerobic conditions.

CVs using a range of high scan rates between 0.2 and 5 V·s$^{-1}$ were also performed in the potential range between –0.2 to +0.6 V (*vs.* Ag/AgCl), as shown in Figure S26. The "blunt" shape of the PEDOT thin film voltammogram at high scan rates is indicative of equivalent series resistance (ESR) due the resistance of the polymer film.[67,68] The PEDOT thin film remains doped over a very wide potential range (in comparison to the PEDOT:PSS film) and therefore has high conductivity even at negative potentials. Indeed, the conductivity of the PEDOT thin film at –0.62 V is still greater than the maximum conductivity of the PEDOT:PSS film at positive potentials where the polymer is fully doped. At high scan rates, the PEDOT thin film displays a more ideal capacitive behaviour due to the polymer films high intrinsic conductivity and wide conductance window.

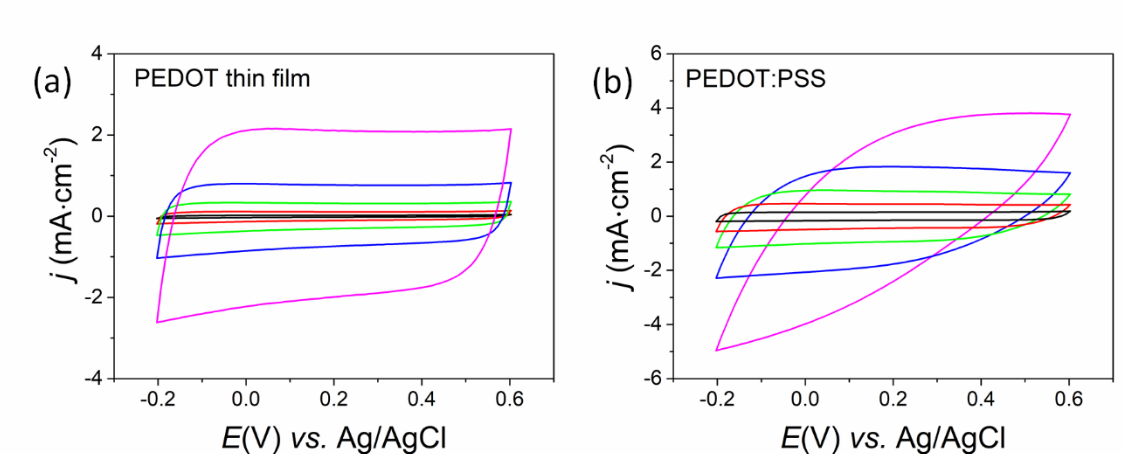


**Figure S26.** CV of (a) a PEDOT thin film prepared by interfacial electrosynthesis and (b) PEDOT:PSS film immobilised on ITO working electrodes in PBS solution at various scan rates (0.2, 0.5, 1, 2 and 5 $V{\cdot}s^{-1}$). CVs were obtained under aerobic conditions.

## Section S8. Biocompatibility studies using PEDOT thin films

### 8.1 Biocompatibility studies: detailed sample preparation

For control experiments, PEDOT:PSS films were prepared by drop-casting a volume of PEDOT:PSS ink (1.1% in $H_2O$, surfactant-free, high-conductivity grade) onto cell culture treated plastic wear. The PEDOT:PSS film was dried overnight in oven at 60 °C.

PEDOT thin films prepared by interfacial electrosynthesis were allowed to attach to the surface of a cell culture treated plastic wear and washed 3 times with 1 × Phosphate buffer saline (PBS) to remove any residual species. Films were sterilized with 30 min of ultraviolet light (UV) exposure. Following sterilization, a group of samples were coated with 10μg/mL collagen in PBS solution for 2 hours at 37 °C. Residual collagen solution was removed.

An adherent cell line of normal human Retina Pigment Epithelium (hTERT RPE-1) of passage number <10 was used for this study. Prior to seeding on the PEDOT films, hTERT RPE-1 cells were grown under normal cell culture conditions at 37 °C and 5% $CO_2$ in Dulbecco's Modified Eagle's Medium (DMEM) supplemented with 10% fetal bovine serum (FBS), 1% penicillin/streptomycin, 1% GlutaMAX, and sodium bicarbonate until they reached 80% confluency. Afterwards, cells were trypsinised, counted, and diluted to a concentration of 5,000 cells/mL. Cells were maintained in culture for 48 hours then stained with fixable dead cell labelling dye following manufacturer's instructions (Zombie green) and fixed with 4% paraformaldehyde solution (PFA) / 4% sucrose/ PBS for 15 min at RT, washed with 1 × PBS with 0.2% Triton-X for 10 min at RT, and blocked with 10% FBS /1× PBS (blocking solution (BS)) for 1h at RT. Afterward, cells were incubated with anti-actin FITCE conjugated dye diluted in BS for 1 hr at RT. After 3 × 5min washes with 1 × PBS, the cells were counterstained with 4′,6-diamidino-2-phenylindole (DAPI).

## 8.2 PSS severely hampers the biocompatibility and integrity of implantable biodevices

In both biosensors and tissue engineering applications, PEDOT:PSS has been employed as a conductive substrate functionalised by target recognising biological macromolecules.[58,69] However, the unsatisfactory long-term stability of these systems in biological environments, where biomolecules have evolved to function, can contribute to sensor or tissue engineer platform instability. In the area of implantable biomedical devices, where device integrity is an absolute requirement for their long-term function, deterioration in performance is typically due to either the delamination and dissolution effects of PSS[70,71] or localised pH alterations by PSS that contribute to biological macromolecule denaturation.[72,73] Additionally, PEDOT:PSS layers are frequently functioning as part of a composite structure, where strong adhesion between materials are required for optimum behaviour.[74] Accordingly, significant efforts have been undertaken to mitigate against these detrimental effects, using post deposition treatments to remove surfactant, like acid washing[75] or chemical capping, for example with the GOPS crosslinker,[47] being employed to increase the stability. The harshness of removal protocols in systems utilising fragile biomolecules or the adverse effects of conductivity due to chemical capping identifies an unmet need for alternative manufacturing processes to avail of the inherent advantage of PEDOT without the troublesome contributions of PSS.

The retinal pigment epithelium (RPE) layer is a monolayer of cells that function as vegetative regulators of overlying photoreceptor cells and as a selective barrier within the retina. They are essential for photoreceptor cell health, function and maintenance[76] and their dysregulation has been identified as an underlying pathology in various eye diseases, including macular degeneration.[77–79] A truly biocompatible substrate should provide an environment where cells are not only assessed for cytotoxic behaviour but are also evaluated against their other indicators of cell health, like their proliferation activity and morphological properties like cell adhesion and spreading.[80] In this context, PEDOT has favourable optical, biocompatible

and electrical properties make is explored in this work (see Figure 4 in main text) as a substrate to facilitate cell growth towards applications as a transducer of electrical signals through photoreceptor cells to the optic nerve.

## Supplementary references


(1) Gschwend, G. C.; Olaya, A.; Peljo, P.; Girault, H. H. Structure and Reactivity of the Polarised Liquid–Liquid Interface: What We Know and What We Do Not. *Curr. Opin. Electrochem.* **2020**, *19*, 137–143. https://doi.org/10.1016/j.coelec.2019.12.002.

(2) Suárez-Herrera, M. F.; Scanlon, M. D. On the Non-Ideal Behaviour of Polarised Liquid-Liquid Interfaces. *Electrochim. Acta* **2019**, *328*, 1–9. https://doi.org/10.1016/j.electacta.2019.135110.

(3) Samec, Z. Dynamic Electrochemistry at the Interface between Two Immiscible Electrolytes. *Electrochim. Acta* **2012**, *84*, 21–28. https://doi.org/10.1016/j.electacta.2012.03.118.

(4) Peljo, P.; Girault, H. H. Liquid/Liquid Interfaces, Electrochemistry At. In *Encyclopedia of Analytical Chemistry*; John Wiley & Sons, Ltd: Chichester, UK, 2012. https://doi.org/10.1002/9780470027318.a5306.pub2.

(5) Samec, Z. Electrochemistry at the Interface between Two Immiscible Electrolyte Solutions (IUPAC Technical Report). *Pure Appl. Chem.* **2004**, *76* (12), 2147–2180. https://doi.org/10.1351/pac200476122147.

(6) McQuin, C.; Goodman, A.; Chernyshev, V.; Kamentsky, L.; Cimini, B. A.; Karhohs, K. W.; Doan, M.; Ding, L.; Rafelski, S. M.; Thirstrup, D.; Wiegraebe, W.; Singh, S.; Becker, T.; Caicedo, J. C.; Carpenter, A. E. CellProfiler 3.0: Next-Generation Image Processing for Biology. *PLoS Biol.* **2018**, *16* (7), 1–17. https://doi.org/10.1371/journal.pbio.2005970.

(7) Smirnov, E.; Peljo, P.; Scanlon, M. D.; Girault, H. H. Gold Nanofilm Redox Catalysis for Oxygen Reduction at Soft Interfaces. *Electrochim. Acta* **2016**, *197*, 362–373. https://doi.org/10.1016/j.electacta.2015.10.104.

(8) Buchanan, C. A.; Ko, E.; Cira, S.; Balasubramanian, M.; Goldsmith, B. R.; Singh, N. Structures and Free Energies of Cerium Ions in Acidic Electrolytes. *Inorg. Chem.* **2020**, *59* (17), 12552–12563. https://doi.org/10.1021/acs.inorgchem.0c01645.

(9) Hotta, H.; Akagi, N.; Sugihara, T.; Ichikawa, S.; Osakai, T. Electron-Conductor Separating Oil-Water (ECSOW) System: A New Strategy for Characterizing Electron-Transfer Processes at the Oil/Water Interface. *Electrochem. commun.* **2002**, *4* (5), 472–477. https://doi.org/10.1016/S1388-2481(02)00343-0.

(10) Hotta, H.; Ichikawa, S.; Sugihara, T.; Osakai, T. Clarification of the Mechanism of Interfacial Electron-Transfer Reaction between Ferrocene and Hexacyanoferrate(III) by Digital Simulation of Cyclic Voltammograms. *J. Phys. Chem. B* **2003**, *107* (36), 9717–9725. https://doi.org/10.1021/jp035058p.

(11) Gamero-Quijano, A.; Molina-Osorio, A. F.; Peljo, P.; Scanlon, M. D. Closed Bipolar Electrochemistry in a Four-Electrode Configuration. *Phys. Chem. Chem. Phys.* **2019**, *21* (19), 9627–9640. https://doi.org/10.1039/C9CP00774A.

(12) Olaya, A. J.; Ge, P.; Girault, H. H. Ion Transfer across the Water|trifluorotoluene Interface. *Electrochem. commun.* **2012**, *19* (1), 101–104. https://doi.org/10.1016/j.elecom.2012.03.010.

(13) Bard, A. J.; Faulkner, L. R. *Electrochemical Methods: Fundamentals and Applications, 2nd Edition*, 2nd ed.; John Wiley & Sons, INC, 2000.

(14) Gamero-Quijano, A.; Herzog, G.; Scanlon, M. D. Bioelectrochemistry of Cytochrome c in a Closed Bipolar Electrochemical Cell. *Electrochem. commun.* **2019**, *109* (November), 106600. https://doi.org/10.1016/j.elecom.2019.106600.

(15) Hölzle, M. H.; Retter, U.; Kolb, D. M. The Kinetics of Structural Changes in Cu Adlayers on Au(111). *J. Electroanal. Chem.* **1994**, *371* (1–2), 101–109. https://doi.org/10.1016/0022-0728(93)03235-H.

(16) Otero, T. F.; Grande, H.; Rodrîguez, J. Conformational Relaxation during Polypyrrole Oxidation: From Experiment to Theory. *Electrochim. Acta* **1996**, *41* (11–12), 1863–1869. https://doi.org/10.1016/0013-4686(96)86826-5.
(17) Herzog, G. Recent Developments in Electrochemistry at the Interface between Two Immiscible Electrolyte Solutions for Ion Sensing. *Analyst* **2015**, *140* (12), 3888–3896. https://doi.org/10.1039/c5an00601e.
(18) Fermín, D. J.; Ding, Z.; Duong, H. D.; Brevet, P.-F.; Girault, H. H. Photoinduced Electron Transfer at Liquid/Liquid Interfaces. 1. Photocurrent Measurements Associated with Heterogeneous Quenching of Zinc Porphyrins. *J. Phys. Chem. B* **1998**, *102* (50), 10334–10341. https://doi.org/10.1021/jp983196m.
(19) Pereira, C. M.; Martins, A.; Rocha, M.; Silva, C. J.; Silva, F. Differential Capacitance of Liquid/Liquid Interfaces: Effect of Electrolytes Present in Each Phase. *J. Chem. Soc. Faraday Trans.* **1994**, *90* (1), 143–148. https://doi.org/10.1039/ft9949000143.
(20) Slevin, C. J.; Malkia, A.; Liljeroth, P.; Toiminen, M.; Kontturi, K. Electrochemical Characterization of Polyelectrolyte Multilayers Deposited at Liquid-Liquid Interfaces. *Langmuir* **2003**, *19* (4), 1287–1294. https://doi.org/10.1021/la0266841.
(21) Garreau, S.; Duvail, J. L.; Louarn, G. Spectroelectrochemical Studies of Poly(3,4-Ethylenedioxythiophene) in Aqueous Medium. *Synth. Met.* **2002**, *125* (3), 325–329. https://doi.org/10.1016/S0379-6779(01)00397-6.
(22) Nuraje, N.; Su, K.; Yang, N. I.; Matsui, H. Liquid / Liquid Interfacial Polymerization To Grow Single Crystalline Nanoneedles. *ACS Nano* **2008**, *2* (3), 502–506. https://doi.org/10.1021/nn7001536.
(23) Bubnova, O.; Khan, Z. U.; Malti, A.; Braun, S.; Fahlman, M.; Berggren, M.; Crispin, X. Optimization of the Thermoelectric Figure of Merit in the Conducting Polymer Poly(3,4-Ethylenedioxythiophene). *Nat. Mater.* **2011**, *10* (6), 429–433. https://doi.org/10.1038/nmat3012.
(24) Wieland, M.; Malacrida, C.; Yu, Q.; Schlewitz, C.; Scapinello, L.; Penoni, A.; Ludwigs, S. Conductance and Spectroscopic Mapping of EDOT Polymer Films upon Electrochemical Doping. *Flex. Print. Electron.* **2020**, *5* (1). https://doi.org/10.1088/2058-8585/ab76e0.
(25) Massonnet, N.; Carella, A.; Jaudouin, O.; Rannou, P.; Laval, G.; Celle, C.; Simonato, J. P. Improvement of the Seebeck Coefficient of PEDOT:PSS by Chemical Reduction Combined with a Novel Method for Its Transfer Using Free-Standing Thin Films. *J. Mater. Chem. C* **2014**, *2* (7), 1278–1283. https://doi.org/10.1039/c3tc31674b.
(26) Łapkowski, M.; Proń, A. Electrochemical Oxidation of Poly(3,4-Ethylenedioxythiophene) - `in Situ' Conductivity and Spectroscopic Investigations. *Synth. Met.* **2000**, *110* (1), 79–83. https://doi.org/10.1016/S0379-6779(99)00271-4.
(27) Garreau, S.; Louarn, G.; Buisson, J. P.; Froyer, G.; Lefrant, S. In Situ Spectroelectrochemical Raman Studies of Poly(3,4-Ethylenedioxythiophene) (PEDT). *Macromolecules* **1999**, *32* (20), 6807–6812. https://doi.org/10.1021/ma9905674.
(28) Im, S. G.; Gleason, K. K. Systematic Control of the Electrical Conductivity of Poly(3,4- Ethylenedioxythiophene) via Oxidative Chemical Vapor Deposition. *Macromolecules* **2007**, *40* (18), 6552–6556. https://doi.org/10.1021/ma0628477.
(29) Wang, X.; Lee, S.; Wang, M.; Zhao, J.; Zhang, X.; Sun, L.; Shao-Horn, Y.; Dincă, M.; Lee, D.; Gleason, K. K.; Palacios, T. High Electrical Conductivity and Carrier Mobility in OCVD PEDOT Thin Films by Engineered Crystallization and Acid Treatment. *Sci. Adv.* **2018**, *4* (9), eaat5780. https://doi.org/10.1126/sciadv.aat5780.
(30) Gueye, M. N.; Carella, A.; Massonnet, N.; Yvenou, E.; Brenet, S.; Faure-Vincent, J.; Pouget, S.; Rieutord, F.; Okuno, H.; Benayad, A.; Demadrille, R.; Simonato, J. P. Structure and Dopant Engineering in PEDOT Thin Films: Practical Tools for a

Dramatic Conductivity Enhancement. *Chem. Mater.* **2016**, *28* (10), 3462–3468. https://doi.org/10.1021/acs.chemmater.6b01035.
(31) Howden, R. M.; McVay, E. D.; Gleason, K. K. OCVD Poly(3,4-Ethylenedioxythiophene) Conductivity and Lifetime Enhancement via Acid Rinse Dopant Exchange. *J. Mater. Chem. A* **2013**, *1* (4), 1334–1340. https://doi.org/10.1039/c2ta00321j.
(32) Lee, S.; Paine, D. C.; Gleason, K. K. Heavily Doped Poly(3,4-Ethylenedioxythiophene) Thin Films with High Carrier Mobility Deposited Using Oxidative CVD: Conductivity Stability and Carrier Transport. *Adv. Funct. Mater.* **2014**, *24* (45), 7187–7196. https://doi.org/10.1002/adfm.201401282.
(33) Reynolds, J. R.; Thompson, B. C.; Skotheim, T. A. *Conjugated Polymers: Properties, Processing, and Applications*; CRC Press, 2019, 2019.
(34) Shi, W.; Yao, Q.; Qu, S.; Chen, H.; Zhang, T.; Chen, L. Micron-Thick Highly Conductive Pedot Films Synthesized via Self-Inhibited Polymerization: Roles of Anions. *NPG Asia Mater.* **2017**, *9* (7). https://doi.org/10.1038/am.2017.107.
(35) Khan, M. A.; Armes, S. P.; Perruchot, C.; Ouamara, H.; Chehimi, M. M.; Greaves, S. J.; Watts, J. F. Surface Characterization of Poly(3,4-Ethylenedioxythiophene)-Coated Latexes by X-Ray Photoelectron Spectroscopy. *Langmuir* **2000**, *16* (9), 4171–4179. https://doi.org/10.1021/la991390+.
(36) Crispin, X.; Marciniak, S.; Osikowicz, W.; Zotti, G.; Denier Van Der Gon, A. W.; Louwet, F.; Fahlman, M.; Groenendaal, L.; De Schryver, F.; Salaneck, W. R. Conductivity, Morphology, Interfacial Chemistry, and Stability of Poly(3,4-Ethylene Dioxythiophene)-Poly(Styrene Sulfonate): A Photoelectron Spectroscopy Study. *J. Polym. Sci. Part B Polym. Phys.* **2003**, *41* (21), 2561–2583. https://doi.org/10.1002/polb.10659.
(37) Bubnova, O.; Khan, Z. U.; Malti, A.; Braun, S.; Fahlman, M.; Berggren, M.; Crispin, X. Optimization of the Thermoelectric Figure of Merit in the Conducting Polymer Poly(3,4-Ethylenedioxythiophene). *Nat. Mater.* **2011**, *10* (6), 429–433. https://doi.org/10.1038/nmat3012.
(38) Shi, W.; Zhao, T.; Xi, J.; Wang, D.; Shuai, Z. Unravelling Doping Effects on PEDOT at the Molecular Level: From Geometry to Thermoelectric Transport Properties. *J. Am. Chem. Soc.* **2015**, *137* (40), 12929–12938. https://doi.org/10.1021/jacs.5b06584.
(39) Bhattacharyya, D.; Gleason, K. K. Single-Step Oxidative Chemical Vapor Deposition of −COOH Functional Conducting Copolymer and Immobilization of Biomolecule for Sensor Application. *Chem. Mater.* **2011**, *23* (10), 2600–2605. https://doi.org/10.1021/cm2002397.
(40) Goubard, F.; Aubert, P. H.; Boukerma, K.; Pauthe, E.; Chevrot, C. Elaboration of Nanohybrid Materials by Photopolymerisation of 3,4-Ethylenedioxythiophene on TiO2. *Chem. Commun.* **2008**, No. 27, 3139–3141. https://doi.org/10.1039/b800754c.
(41) Yang, Y.; Deng, H.; Fu, Q. Recent Progress on PEDOT:PSS Based Polymer Blends and Composites for Flexible Electronics and Thermoelectric Devices. *Mater. Chem. Front.* **2020**, *4* (11), 3130–3152. https://doi.org/10.1039/d0qm00308e.
(42) Marquez, A. V.; Mcevoy, N. Organic Electrochemical Transistors ( OECTs ) toward Flexible and Wearable Bioelectronics. **2020**, 1–25.
(43) Spencer, A. R.; Primbetova, A.; Koppes, A. N.; Koppes, R. A.; Fenniri, H.; Annabi, N. Electroconductive Gelatin Methacryloyl-PEDOT:PSS Composite Hydrogels: Design, Synthesis, and Properties. *ACS Biomater. Sci. Eng.* **2018**, *4* (5), 1558–1567. https://doi.org/10.1021/acsbiomaterials.8b00135.
(44) Cameron, J.; Skabara, P. J. The Damaging Effects of the Acidity in PEDOT:PSS on Semiconductor Device Performance and Solutions Based on Non-Acidic Alternatives.

*Mater. Horizons* **2020**, *7* (7), 1759–1772. https://doi.org/10.1039/c9mh01978b.
(45) Grossiord, N.; Kroon, J. M.; Andriessen, R.; Blom, P. W. M. Degradation Mechanisms in Organic Photovoltaic Devices. *Org. Electron.* **2012**, *13* (3), 432–456. https://doi.org/10.1016/j.orgel.2011.11.027.
(46) Stöcker, T.; Köhler, A.; Moos, R. Why Does the Electrical Conductivity in PEDOT:PSS Decrease with PSS Content? A Study Combining Thermoelectric Measurements with Impedance Spectroscopy. *J. Polym. Sci. Part B Polym. Phys.* **2012**, *50* (14), 976–983. https://doi.org/10.1002/polb.23089.
(47) Håkansson, A.; Han, S.; Wang, S.; Lu, J.; Braun, S.; Fahlman, M.; Berggren, M.; Crispin, X.; Fabiano, S. Effect of (3-Glycidyloxypropyl)Trimethoxysilane (GOPS) on the Electrical Properties of PEDOT:PSS Films. *J. Polym. Sci. Part B Polym. Phys.* **2017**, *55* (10), 814–820. https://doi.org/10.1002/polb.24331.
(48) Airaghi Leccardi, M. J. I.; Chenais, N. A. L.; Ferlauto, L.; Kawecki, M.; Zollinger, E. G.; Ghezzi, D. Photovoltaic Organic Interface for Neuronal Stimulation in the Near-Infrared. *Commun. Mater.* **2020**, *1* (1), 1–13. https://doi.org/10.1038/s43246-020-0023-4.
(49) Stavrinidou, E.; Leleux, P.; Rajaona, H.; Khodagholy, D.; Rivnay, J.; Lindau, M.; Sanaur, S.; Malliaras, G. G. Direct Measurement of Ion Mobility in a Conducting Polymer. *Adv. Mater.* **2013**, *25* (32), 4488–4493. https://doi.org/10.1002/adma.201301240.
(50) Cameron, J.; Skabara, P. J. Materials Horizons The Damaging Effects of the Acidity in PEDOT : PSS Solutions Based on Non-Acidic Alternatives. **2020**, 1759–1772. https://doi.org/10.1039/c9mh01978b.
(51) Gueye, M. N.; Carella, A.; Faure-Vincent, J.; Demadrille, R.; Simonato, J. P. Progress in Understanding Structure and Transport Properties of PEDOT-Based Materials: A Critical Review. *Prog. Mater. Sci.* **2020**, *108* (September 2019), 100616. https://doi.org/10.1016/j.pmatsci.2019.100616.
(52) Cho, B.; Park, K. S.; Baek, J.; Oh, H. S.; Koo Lee, Y. E.; Sung, M. M. Single-Crystal Poly(3,4-Ethylenedioxythiophene) Nanowires with Ultrahigh Conductivity. *Nano Lett.* **2014**, *14* (6), 3321–3327. https://doi.org/10.1021/nl500748y.
(53) Guo, Z.; Panda, D. K.; Maity, K.; Lindsey, D.; Parker, T. G.; Albrecht-Schmitt, T. E.; Barreda-Esparza, J. L.; Xiong, P.; Zhou, W.; Saha, S. Modulating the Electrical Conductivity of Metal-Organic Framework Films with Intercalated Guest π-Systems. *J. Mater. Chem. C* **2016**, *4* (5), 894–899. https://doi.org/10.1039/c5tc02232k.
(54) Seng, S.; Shinpei, T.; Yoshihiko, I.; Masakazu, K. Development of a Handmade Conductivity Measurement Device for a Thin-Film Semiconductor and Its Application to Polypyrrole. *J. Chem. Educ.* **2014**, *91* (11), 1971–1975. https://doi.org/10.1021/ed500287q.
(55) Zotti, G.; Zecchin, S.; Schiavon, G.; Groenendaal, L. B. Conductive and Magnetic Properties of 3,4-Dimethoxy- and 3,4-Ethylenedioxy-Capped Polypyrrole and Polythiophene. *Chem. Mater.* **2000**, *12* (10), 2996–3005. https://doi.org/10.1021/cm000400l.
(56) Zotti, G.; Zecchin, S.; Schiavon, G.; Louwet, F.; Groenendaal, L.; Crispin, X.; Osikowicz, W.; Salaneck, W.; Fahlman, M. Electrochemical and XPS Studies toward the Role of Monomeric and Polymeric Sulfonate Counterions in the Synthesis, Composition, and Properties of Poly(3,4-Ethylenedioxythiophene). *Macromolecules* **2003**, *36* (9), 3337–3344. https://doi.org/10.1021/ma021715k.
(57) Tang, K.; Miao, W.; Guo, S. Crosslinked PEDOT:PSS Organic Electrochemical Transistors on Interdigitated Electrodes with Improved Stability. *ACS Appl. Polym. Mater.* **2021**, *3* (3), 1436–1444. https://doi.org/10.1021/acsapm.0c01292.

(58) Rivnay, J.; Inal, S.; Salleo, A.; Owens, R. M.; Berggren, M.; Malliaras, G. G. Organic Electrochemical Transistors. *Nat. Rev. Mater.* **2018**, *3*, 1–41. https://doi.org/10.1038/natrevmats.2017.86.
(59) Kim, Y.; Lim, T.; Kim, C. H.; Yeo, C. S.; Seo, K.; Kim, S. M.; Kim, J.; Park, S. Y.; Ju, S.; Yoon, M. H. Organic Electrochemical Transistor-Based Channel Dimension-Independent Single-Strand Wearable Sweat Sensors. *NPG Asia Mater.* **2018**, *10* (11), 1086–1095. https://doi.org/10.1038/s41427-018-0097-3.
(60) Heinze, J. J.; Frontana-Uribe, B. A.; Ludwigs, S. Electrochemistry of Conducting Polymers—Persistent Models and New Concepts. *Chem. Rev.* **2010**, *110* (8), 4724–4771. https://doi.org/10.1021/cr900226k.
(61) Bernards, D. A.; Malliaras, G. G. Steady-State and Transient Behavior of Organic Electrochemical Transistors. *Adv. Funct. Mater.* **2007**, *17* (17), 3538–3544. https://doi.org/10.1002/adfm.200601239.
(62) Ofer, D.; Crooks, R. M.; Wrighton, M. S. Potential Dependence of the Conductivity of Highly Oxidized Poly Thiophenes, Polypyrroles, and Poly Aniline: Finite Windows of High Conductivity. *J. Am. Chem. Soc.* **1990**, *112* (22), 7869–7879. https://doi.org/10.1021/ja00178a004.
(63) Nishizawa, M.; Ise, T.; Koshika, H.; Itoh, T.; Uchida, I. Electrochemical In-Situ Conductivity Measurements for Thin Film of Li(1-x)Mn2O4 Spinel. *Chem. Mater.* **2000**, *12* (5), 1367–1371. https://doi.org/10.1021/cm990696z.
(64) Kingsborough, R. P.; Swager, T. M. Polythiophene Hybrids of Transition-Metal Bis(Salicylidenimine)s: Correlation between Structure and Electronic Properties. *J. Am. Chem. Soc.* **1999**, *121* (38), 8825–8834. https://doi.org/10.1021/ja991285m.
(65) Heinze, J.; Frontana-Uribe, B. A.; Ludwigs, S. Electrochemistry of Conducting Polymers-Persistent Models and New Concepts. *Chem. Rev.* **2010**, *110* (8), 4724–4771. https://doi.org/10.1021/cr900226k.
(66) Morvant, M. C.; Reynolds, J. R. In Situ Conductivity Studies of Poly(3,4-Ethylenedioxythiophene). *Synth. Met.* **1998**, *92* (1), 57–61. https://doi.org/10.1016/s0379-6779(98)80023-4.
(67) Boonpakdee, D.; Guajardo Yévenes, C. F.; Surareungchai, W.; La-o-vorakiat, C. Exploring Non-Linearities of Carbon-Based Microsupercapacitors from an Equivalent Circuit Perspective. *J. Mater. Chem. A* **2018**, *6* (16), 7162–7167. https://doi.org/10.1039/C8TA01995A.
(68) Mathis, T. S.; Kurra, N.; Wang, X.; Pinto, D.; Gogotsi, Y.; Mathis, T. S.; Kurra, N.; Wang, X.; Pinto, D.; Simon, P.; Stor-, E. Energy Storage Data Reporting in Perspective-Guidelines for Interpreting the Performance of Electrochemical Energy Storage Systems To Cite This Version : HAL Id : Hal-02519795. **2020**. https://doi.org/10.1002/aenm.201902007.
(69) Guo, B.; Ma, P. X. Conducting Polymers for Tissue Engineering. *Biomacromolecules* **2018**, *19* (6), 1764–1782. https://doi.org/10.1021/acs.biomac.8b00276.
(70) Green, R. A.; Hassarati, R. T.; Bouchinet, L.; Lee, C. S.; Cheong, G. L. M.; Yu, J. F.; Dodds, C. W.; Suaning, G. J.; Poole-Warren, L. A.; Lovell, N. H. Substrate Dependent Stability of Conducting Polymer Coatings on Medical Electrodes. *Biomaterials* **2012**, *33* (25), 5875–5886. https://doi.org/10.1016/j.biomaterials.2012.05.017.
(71) Cui, X. T.; Zhou, D. D. Poly (3,4-Ethylenedioxythiophene) for Chronic Neural Stimulation. *IEEE Trans. Neural Syst. Rehabil. Eng.* **2007**, *15* (4), 502–508. https://doi.org/10.1109/TNSRE.2007.909811.
(72) Wan, A. M. D.; Schur, R. M.; Ober, C. K.; Fischbach, C.; Gourdon, D.; Malliaras, G. G. Electrical Control of Protein Conformation. *Adv. Mater.* **2012**, *24* (18), 2501–2505. https://doi.org/10.1002/adma.201200436.

(73) E C Williams, P A Janmey, J. D. F. and D. F. M. Conformational States of Fibronectin. *J. Biol. Chem.* **1982**, *257* (24), 14973–14978. https://doi.org/10.1016/S0021-9258.
(74) Liu, T.; Sun, L.; Dong, X.; Jiang, Y.; Wang, W.; Xie, C.; Zeng, W.; Liu, Y.; Qin, F.; Hu, L.; Zhou, Y. Low-Work-Function PEDOT Formula as a Stable Interlayer and Cathode for Organic Solar Cells. *Adv. Funct. Mater.* **2021**, *2107250*, 1–8. https://doi.org/10.1002/adfm.202107250.
(75) Liu, D.; Rahman, M. M.; Ge, C.; Kim, J.; Lee, J. J. Highly Stable and Conductive PEDOT:PSS/Graphene Nanocomposites for Biosensor Applications in Aqueous Medium. *New J. Chem.* **2017**, *41* (24), 15458–15465. https://doi.org/10.1039/c7nj03330c.
(76) Ballios, B. G.; Clarke, L.; Coles, B. L. K.; Shoichet, M. S.; Van Der Kooy, D. The Adult Retinal Stem Cell Is a Rare Cell in the Ciliary Epithelium Whose Progeny Can Differentiate into Photoreceptors. *Biol. Open* **2012**, *1* (3), 237–246. https://doi.org/10.1242/bio.2012027.
(77) Forest, D. L.; Johnson, L. V.; Clegg, D. O. Cellular Models and Therapies for Age-Related Macular Degeneration. *DMM Dis. Model. Mech.* **2015**, *8* (5), 421–427. https://doi.org/10.1242/dmm.017236.
(78) Somasundaran, S.; Constable, I. J.; Mellough, C. B.; Carvalho, L. S. Retinal Pigment Epithelium and Age-Related Macular Degeneration: A Review of Major Disease Mechanisms. *Clin. Exp. Ophthalmol.* **2020**, *48* (8), 1043–1056. https://doi.org/10.1111/ceo.13834.
(79) Sharma, R.; Bose, D.; Maminishkis, A.; Bharti, K. Retinal Pigment Epithelium Replacement Therapy for Age-Related Macular Degeneration: Are We There Yet? *Annu. Rev. Pharmacol. Toxicol.* **2020**, *60*, 553–572. https://doi.org/10.1146/annurev-pharmtox-010919-023245.
(80) Anderson, J. M. Biocompatibility. *Polym. Sci. A Compr. Ref. 10 Vol. Set* **2012**, *9*, 363–383. https://doi.org/10.1016/B978-0-444-53349-4.00229-6.
